# Prevention of acquired sensorineural hearing loss in mice by in vivo *Htra2* gene editing

**DOI:** 10.1186/s13059-021-02311-4

**Published:** 2021-03-22

**Authors:** Xi Gu, Daqi Wang, Zhijiao Xu, Jinghan Wang, Luo Guo, Renjie Chai, Genglin Li, Yilai Shu, Huawei Li

**Affiliations:** 1 ENT institute and Department of Otorhinolaryngology, Eye & ENT Hospital, State Key Laboratory of Medical Neurobiology and MOE Frontiers Center for Brain Science, Fudan University, Shanghai, 200031 China; 2 Institutes of Biomedical Sciences, Fudan University, Shanghai, 200032 China; 3 Department of Otolaryngology, the First Affiliated Hospital of Fujian Medical University, Fuzhou, 350005 China; 4 NHC Key Laboratory of Hearing Medicine (Fudan University), Shanghai, 200031 China; 5 Key Laboratory for Developmental Genes and Human Disease, Ministry of Education, Institute of Life Sciences, Jiangsu Province High-Tech Key Laboratory for Bio-Medical Research, Southeast University, Nanjing, 210096 China; 6 Co-Innovation Center of Neuroregeneration, Nantong University, Nantong, 226001 China; 7 Institute for Stem Cell and Regeneration, Chinese Academy of Science, Beijing, China; 8 The Institutes of Brain Science and the Collaborative Innovation Center for Brain Science, Fudan University, Shanghai, 200032 China

**Keywords:** CRISPR/Cas9, Genome editing, Omi/HtrA2, Ototoxic deafness, Aminoglycoside antibiotics, Hair cell, Cochlea, Anti-apoptosis

## Abstract

**Background:**

Aging, noise, infection, and ototoxic drugs are the major causes of human acquired sensorineural hearing loss, but treatment options are limited. CRISPR/Cas9 technology has tremendous potential to become a new therapeutic modality for acquired non-inherited sensorineural hearing loss. Here, we develop CRISPR/Cas9 strategies to prevent aminoglycoside-induced deafness, a common type of acquired non-inherited sensorineural hearing loss, via disrupting the *Htra2* gene in the inner ear which is involved in apoptosis but has not been investigated in cochlear hair cell protection.

**Results:**

The results indicate that adeno-associated virus (AAV)-mediated delivery of CRISPR/SpCas9 system ameliorates neomycin-induced apoptosis, promotes hair cell survival, and significantly improves hearing function in neomycin-treated mice. The protective effect of the AAV–CRISPR/Cas9 system in vivo is sustained up to 8 weeks after neomycin exposure. For more efficient delivery of the whole CRISPR/Cas9 system, we also explore the AAV–CRISPR/SaCas9 system to prevent neomycin-induced deafness. The in vivo editing efficiency of the SaCas9 system is 1.73% on average. We observed significant improvement in auditory brainstem response thresholds in the injected ears compared with the non-injected ears. At 4 weeks after neomycin exposure, the protective effect of the AAV–CRISPR/SaCas9 system is still obvious, with the improvement in auditory brainstem response threshold up to 50 dB at 8 kHz.

**Conclusions:**

These findings demonstrate the safe and effective prevention of aminoglycoside-induced deafness via *Htra2* gene editing and support further development of the CRISPR/Cas9 technology in the treatment of non-inherited hearing loss as well as other non-inherited diseases.

## Background

Sensorineural hearing loss (SNHL) is mainly acquired and affects approximately 1.3 billion humans worldwide [1]. It is related to aging, noise, infection, ototoxic drugs, and genetic defects. Compared with inherited factors, non-inherited forms of acquired SNHL account for a larger proportion in the deaf population. However, the treatment options are limited and there have been no proven preventative or regenerative interventions for high-risk individuals so far. Aminoglycosides, such as gentamicin, sisomicin, streptomycin, kanamycin, and neomycin, are broad-spectrum antibiotics that are particularly effective against aerobic gram-negative bacteria. Despite their serious side effect of irreversible hearing loss [2–6], the drugs are used for many life-threatening bacterial infections, including sepsis, tuberculosis, respiratory infections in cystic fibrosis, complex urinary tract infections, and infective endocarditis [7–13]. There is extensive evidence suggesting that oxidative stress-induced apoptosis and necrosis in hair cells of the cochlea, marginal cells, and the stria vascularis [14] underlie drug-induced inner ear damage. Aminoglycoside antibiotics mainly accumulate in the mitochondria and lysosomes of cochlear hair cells causing an increase in oxygen free radicals that in turn induce apoptosis and eventually lead to hearing loss [4, 15–17]. There have been some attempts to protect auditory and vestibular function from aminoglycoside-induced ototoxicity, including reducing the uptake of aminoglycosides in cochlear hair cells and protecting hair cells by ameliorating intracellular cytotoxicity [18]. However, none of these strategies has been approved for the treatment of drug-induced deafness, and thus, there is still tremendous demand to explore novel protective compounds and therapeutic strategies. To develop new therapeutic strategies for acquired non-inherited SNHL, we selected the mouse model of ototoxic deafness caused by aminoglycoside antibiotics.

The *Htra2* gene, which encodes a proapoptotic mitochondrial serine protease (Omi/HtrA2), induces apoptosis by binding to the apoptosis inhibitory protein XIAP (X-linked inhibitor of apoptosis protein) [19, 20]. XIAP is an endogenous inhibitor of caspases [21, 22], while Omi/HtrA2 enhances caspase activity by degrading XIAP and thus participates in the mitochondrial apoptosis pathway [23, 24]. In a previous study [25], we demonstrated a protective role for XIAP against neomycin-induced hearing loss and hair cell loss during the ototoxic-sensitive period. We found that the expression levels of the apoptotic factors *Casp3*, *Casp9*, *Diablo*, and *Htra2* were significantly higher in neomycin-treated cochleae compared to saline controls. Based on these studies, we hypothesized that inhibition of Omi/HtrA2 protects hair cells from aminoglycoside-induced ototoxicity in mice.

The CRISPR/Cas9 technology can be used for targeted gene disruption or repair. Due to its versatility, simplicity, efficiency, and high specificity, the CRISPR/Cas9 technology has been applied for the treatment of Duchenne muscular dystrophy, Hutchinson–Gilford progeria syndrome, Leber congenital amaurosis, and fragile X syndrome in preclinical studies, as well as cancer in a phase 1 clinical trial [26–32]. This advanced technology has also been applied in gene therapy for hereditary hearing loss in mice [33–36], but there have been no reports of the application of this technology in the treatment of acquired non-inherited SNHL in animal models. Therefore, we selected *Htra2* as the target gene and constructed CRISPR/Cas9–*Htra2* knock out systems with the first aim of preventing aminoglycoside-induced hearing loss in mice.

## Results

### Disruption of the Htra2 gene with the SpCas9 system in vitro

**Fig. 1:**
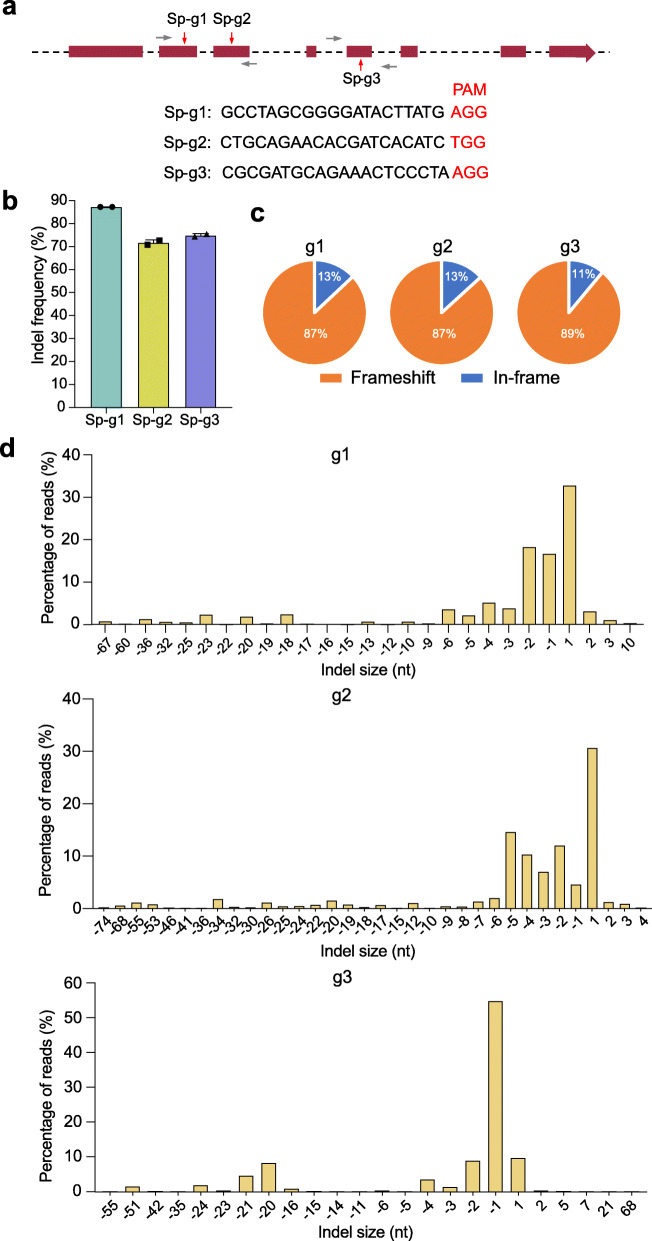
In vitro genome editing with the SpCas9 therapeutic system. **a** Design of the gRNAs. The three gRNA targeting sequences in the *Htra2* gene are listed. PAM sites are marked by red nucleotides. The red arrows indicate the exons targeted by the gRNAs, and the gray arrows indicate the primers. **b** The indel frequency of the gRNAs was tested in HEI-OC1 cells using high-throughput sequencing. Dots represent individual values and bars represent the mean ± SD, *n* = 2. **c** Indels causing in-frame versus frameshift mutations (percentages are shown) in the coding sequence after transfection with SpCas9–gRNA. **d** Indel profiles from HEI-OC1 cells transfected with Sp-g1, Sp-g2, and Sp-g3, respectively. Negative numbers represent deletions, and positive numbers represent insertions. nt nucleotides

In order to disrupt the *Htra2* gene, we designed three guide RNAs (gRNAs) in *Streptococcus pyogenes* Cas9 (SpCas9 with the protospacer-adjacent motif (PAM) sequence of 5′-NGG-3′) system using the DeepHF tools [37] (Fig. 1a). We tested the indel frequency in the *Htra2* gene and the disruption efficiency of the Omi/HtrA2 protein in HEI-OC1 cells. GFP-positive cells were sorted by fluorescence-activated cell sorting (FACS) at 48 h after transfection with SpCas9–gRNA, and the cells were cultured for another 7 days. We then collected the cells for sequencing and western blot (Additional file 1: Fig. S1A). We observed indel frequencies of 87.27 ± 0.02%, 71.72 ± 1.27%, and 74.88 ± 0.77% for the three gRNAs Sp-g1, Sp-g2, and Sp-g3, respectively (Fig. 1b and Additional file 2). The deep sequencing data showed 68, 58, and 69 types of indel mutations for the three gRNAs, respectively, and the major type of mutation was deletions (Fig. 1d and Additional file 3). The proportions of frameshift mutation were 87%, 87%, and 89% for Sp-g1, Sp-g2, and Sp-g3, respectively (Fig. 1c). Western blot showed that the disruption efficiencies of the protein were 86.12%, 32.87%, and 52.26% for Sp-g1, Sp-g2, and Sp-g3, respectively, and these were consistent with the indel efficiencies (Additional file 1: Fig. S1B–C, Additional file 4). Together, these results indicated that the *Htra2* gene can be efficiently edited with the SpCas9 system.

### The Anc80L65-EGFP transduces cochlear hair cells with high efficiency in vitro and in vivo

We selected Anc80L65 as the delivery vector for our in vitro and in vivo experiments because it has been proven to be efficient and safe in mouse hair cells [38]. To test the efficiency of Anc80L65 in transducing cochlear hair cells in vitro and in vivo, we performed viral transduction experiments with Anc80L65-EGFP. After being incubated in DMEM/F12 medium containing Anc80L65-EGFP for 48 h and culturing in DMEM/F12 medium without virus for another 6 days, the transduction efficiencies of the inner hair cells (IHCs) of the apical, middle, and basal turns of the cochlea were 97.7 ± 4.6%, 98.2 ± 3.6%, and 98.3 ± 3.4%, respectively. For outer hair cells (OHCs), the transduction efficiencies of the apical, middle, and basal turns of the cochlea were 93.6 ± 4.8%, 95.5 ± 3.5%, and 96.0 ± 3.1%, respectively (Additional file 1: Fig. S2A). We also performed an in vivo experiment of Anc80L65-EGFP transduction in the inner ear and found that Anc80L65-EGFP transduced 100% of the IHCs and about 90% of the OHCs (96.1 ± 2.2%, 90.9 ± 3.2%, and 88.4 ± 2.6% for the apical, middle, and basal turns, respectively) at 10 days after injection of the virus into the scala media of the inner ear with an injection volume of 500 nl (Additional file 1: Fig. S2B).

### AAV-mediated delivery of Htra2-targeted SpCas9 system in vitro

**Fig. 2:**
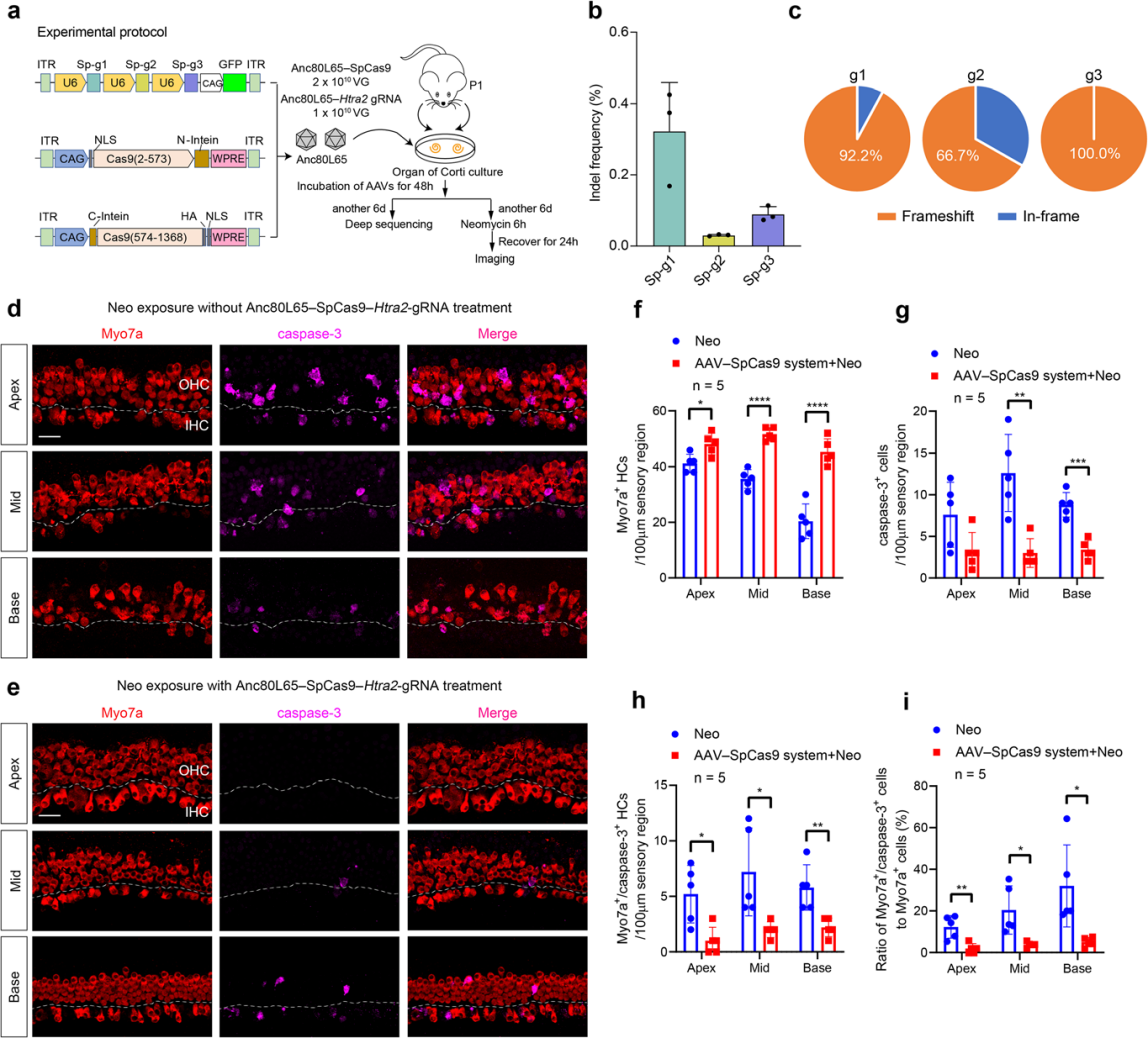
The Anc80L65–SpCas9–*Htra2* gRNA therapeutic system protected hair cells from neomycin-induced injury in vitro. **a** Experimental procedures for the in vitro studies with application of the Anc80L65–SpCas9 therapeutic system. **b** The indel frequency of the gRNAs was measured in the cultured basilar membrane at 8 days post-transduction with the Anc80L65–SpCas9 therapeutic system using high-throughput sequencing. Dots represent individual values, and bars represent the mean ± SD, *n* = 3. **c** Indels causing in-frame versus frameshift mutations in the coding sequence after co-transduction of Anc80L65–SpCas9 and Anc80L65–gRNA. **d** Representative confocal images of neomycin-treated cochlear explants without Anc80L65–SpCas9–*Htra2* gRNA treatment. **e** Representative confocal images of Myo7a and caspase-3 immunofluorescence in neomycin-treated cochlear explants treated with Anc80L65–SpCas9–*Htra2* gRNA for 8 days prior to neomycin exposure. IHC inner hair cell, OHC outer hair cell. Both scale bars (in **d** and **e**) represent 20 μm long and apply to all images. **f**–**h** Comparison of the numbers of Myo7a^+^ hair cells (**f**), caspase-3^+^ cells (**g**), and Myo7a^+^/caspase-3^+^ hair cells (**h**) per 100-μm sensory epithelium region between the neomycin-treated groups with or without Anc80L65–SpCas9–*Htra2* gRNA treatment in the apical, middle, and basal turns of the cochlea. **i** Comparison of the ratio of Myo7a^+^/caspase-3^+^ cells to Myo7a^+^ cells between the neomycin-treated groups with or without Anc80L65–SpCas9–*Htra2* gRNA treatment. Dots represent individual values, and bars represent the mean ± SD in **f**–**i**. For statistical analysis in **f**–**i**, Mann–Whitney *U*-tests, unpaired two-tailed Student’s *t*-tests, or unpaired *t*-tests with Welch’s correction were used as applicable. *p < 0.05, **p < 0.01, ***p < 0.001, ****p < 0.0001. *n* = 5

One advantage of the CRISPR/Cas9 system is that it can edit multiple sites by delivering a single Cas9 enzyme and multiple gRNAs. We concatenated three gRNAs in one plasmid with the purpose of knocking out the *Htra2* gene more efficiently (Fig. 2a). We transfected HEI-OC1 cells with the SpCas9 plasmid and the concatenated gRNA plasmid, collected GFP-positive cells by FACS, isolated DNA from GFP-positive cells, and measured the editing efficiencies of the three gRNAs by high-throughput sequencing (HTS) (Additional file 1: Fig. S3A-E). For the low indel frequency of Sp-g2, we speculated that deletions of large fragments between the target site of Sp-g1 and Sp-g2 might affect the measurement of the indel frequency of Sp-g2 (Additional file 1: Fig. S3F-G, Additional file 4). Off-target tests of the three gRNAs were analyzed in silico using the Cas-OFFinder software [39], and the results demonstrated no obvious indel mutations with fewer than 3 bp mismatches for this SpCas9 system (Additional file 5).

To determine the editing efficiency of the SpCas9 system with three concatenated gRNAs in cultured cochlear explants, Anc80L65 was used to deliver the entire SpCas9 system. In consideration of the limited packaging capacity of AAV (~ 4.8–4.9 kb, including inverted terminal repeats), we employed the split-SpCas9 scheme because it has been shown that it is feasible for SpCas9 to be split at the 573/574 site [40]. In brief, the SpCas9-N-terminal and SpCas9-C-terminal were fused to the corresponding split-intein moieties from *Nostoc punctiforme* (Fig. 2a) and then packaged into AAV2/Anc80L65. The Anc80L65–SpCas9-N-terminal and Anc80L65–SpCas9-C-terminal were mixed in a 1:1 ratio, and Anc80L65–gRNA(g1-g2-g3) was added into the mixture of Anc80L65–SpCas9 at a 1:2 ratio (Fig. 2a). The viral vectors of the CRISPR/SpCas9 system (SpCas9 and gRNA) were applied to the cochlear explants 2 h after culture, and genomic DNA of the whole cochlear tissue, in which the fraction of hair cells was only about 1.5% of the total cells used for DNA sequencing, was isolated after 8 days of culture. The indel frequencies of the three gRNAs (Sp-g1, Sp-g2, and Sp-g3) were 0.32%, 0.03%, and 0.09%, respectively (Fig. 2b, Additional file 2). The most common mutations were frameshift deletions (Fig. 2c).

### The Anc80L65–SpCas9 therapeutic system protects hair cells from neomycin-induced ototoxicity in vitro

To determine whether the Anc80L65–SpCas9 therapeutic system protected hair cells from neomycin-induced ototoxicity, we first performed in vitro experiments. Wang et al. [41] reported that significantly elevated expression of cytoplasmic Omi/HtrA2 triggers cardiomyocyte apoptosis, and they observed that overexpression of cardiac-specific mitochondrial Omi/HtrA2 induced increased myocardial apoptosis [42]. We found that the expression of the Omi/HtrA2 protein was increased after neomycin exposure compared with the normal control group (Additional file 1: Fig. S4). To evaluate the effect of the Anc80L65–SpCas9 therapeutic system on hair cell survival after neomycin exposure in vitro, we applied Anc80L65–SpCas9 and Anc80L65–gRNA to cochlear explants for 8 days prior to neomycin exposure. The neomycin-treated cochlear explants without the Anc80L65–SpCas9 system (SpCas9 + *Htra2* gRNA) (Fig. 2d) exhibited substantial loss of OHCs and partial degeneration of IHCs across the entire cochlea compared with the neomycin-treated cochlear explants pre-treated with the Anc80L65–SpCas9 system (Fig. 2e). There were more hair cells (48.2 ± 3.8, 51.6 ± 2.3, and 45.4 ± 4.7 cells/100 μm for the apical, middle, and basal turns of the cochlea, respectively) in the explants pre-treated with the Anc80L65–SpCas9 system compared to the numbers of hair cells (41.2 ± 3.3, 35.6 ± 3.4, and 20.4 ± 6.2 cells/100 μm in the apical, middle, and basal turns of the cochlea, respectively) in the control group without pre-treatment of the Anc80L65–SpCas9 system (Fig. 2f, Additional file 6). Caspase-mediated apoptosis is a common mechanism for aminoglycoside-induced ototoxicity [43, 44], and caspase-3, one of the primary apoptosis executioner molecules [45, 46], has been widely used to detect apoptosis in hair cells [47–49]. Pre-treatment with Anc80L65–SpCas9 and Anc80L65–gRNA reduced the expression of caspase-3 in the sensory epithelium (treated: 3.2 ± 2.3, 3.0 ± 1.7, and 3.4 ± 1.1 caspase-3-positive cells/100 μm for the apical, middle, and basal turns of the cochlea, respectively; non-treated: 7.6 ± 3.9, 12.6 ± 4.6, and 8.8 ± 1.5 caspase-3-positive cells/100 μm for the apical, middle, and basal turns of the cochlea, respectively), which confirmed the anti-apoptotic effect of the Anc80L65–SpCas9 therapeutic system against neomycin ototoxicity (Fig. 2g–i, Additional file 6). Pre-treatment with Anc80L65–SpCas9 but without Anc80L65–gRNA did not protect hair cells from neomycin-induced damage (Additional file 1: Fig. S5, Additional file 6).

### The in vivo treatment of Anc80L65–SpCas9 system attenuates hair cell loss after neomycin exposure

**Fig. 3:**
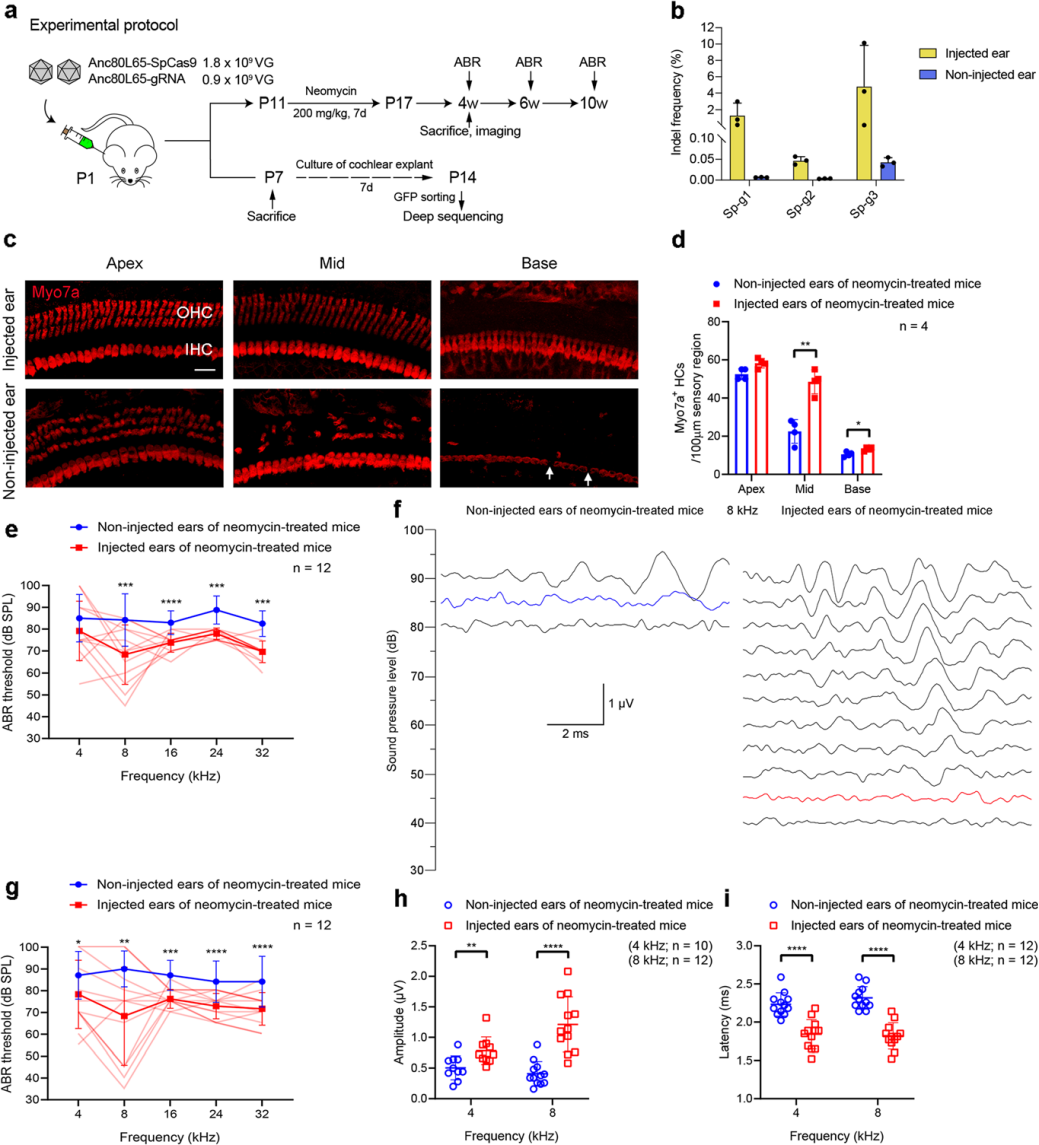
Effects of Anc80L65–SpCas9 therapeutic system on hair cell survival and hearing function in neomycin-exposure mice. **a** Experimental overview. **b** The individual (dot) and mean ± SD (bar) indel frequency of the three gRNAs. *n* = 4. **c** Representative confocal images showing immunofluorescence staining with Myo7a (red) 2 weeks after the last dose of neomycin from an injected cochlea and the contralateral (non-injected) cochlea. IHC inner hair cell, OHC outer hair cell. Arrows indicate the missing IHCs. Scale bar represents 20 μm long and applies to all images. **d** Quantification of cochlear hair cells 2 weeks after the last dose of neomycin. Individual values are shown; bars represent the mean ± SD. Unpaired two-tailed Student’s *t*-tests or Mann–Whitney *U*-tests were used as applicable. **e** Comparison of the ABR thresholds between the non-injected and injected ears 2 weeks after the last dose of neomycin. **f** Families of ABR waveforms recorded at 2 weeks after the last dose of neomycin from the representative non-injected ear and the single best ear injected with Anc80L65–SpCas9 system. The blue trace indicates the threshold of the non-injected ear while the red trace indicates the threshold of the injected ear. The scale bar applies to all traces. **g** Comparison of the ABR thresholds between the non-injected and injected ears 4 weeks after the last dose of neomycin. The lighter red lines in **e** and **g** represent the ABR threshold of individual injected ears. For statistical analysis in **e** and **g**, two-tailed paired *t*-tests or Wilcoxon matched-pairs signed rank tests were used as applicable. **h**, **i** Peak amplitudes (**h**) and latencies (**i**) of ABR wave 1 evoked by 90 dB SPL in the Anc80L65–SpCas9–*Htra2* gRNA-injected ears compared with the non-injected ears 4 weeks after the last dose of neomycin. Unpaired two-tailed Student’s *t*-tests or unpaired *t*-tests with Welch’s correction were used as applicable. Data are presented as the mean ± SD. *p < 0.05, **p < 0.01, ***p < 0.001, ****p < 0.0001

The in vivo editing efficiency detected from the whole basilar membrane was not as high as expected (Additional file 1: Fig. S6), and we speculated that most of the cells in the basilar membrane—in which supporting cells vastly outnumber hair cells—were not transduced by the Anc80L65–SpCas9 system. After sorting GFP-positive cells from the injected cochlea by FACS, the editing efficiency of the three gRNAs was increased by 6.4, 4.8, and 153.3 times (1.32%, 0.05%, and 4.82% for Sp-g1, Sp-g2, and Sp-g3, respectively) (Fig. 3b, Additional file 2).

After neomycin exposure, the OHCs of the mouse cochlea undergo progressive death from the basal to apical turns, followed by partial degeneration of IHCs of the middle and basal turns. To determine the effect of SpCas9–gRNA on hair cell survival in vivo, we injected the Anc80L65–SpCas9 system into the scala media of the inner ear of mice on postnatal day 1 (P1) and isolated the injected and non-injected cochleae at 2 weeks after the last injection of neomycin. Non-injected ears exhibited substantial loss of OHCs and partial degeneration of IHCs compared with injected ears in neomycin-treated mice (Fig. 3c, d, Additional file 6). The survival of hair cells was significantly enhanced in the injected ears, and there were more hair cells in the injected ears (48.5 ± 6.2 and 13.3 ± 1.0 cells/100 μm in the middle and basal turns of the cochlea, respectively) than in the non-injected ears (22.5 ± 6.2 and 10.5 ± 1.3 cells/100 μm in the middle and basal turns of the cochlea, respectively) (Fig. 3d, Additional file 6). These results suggest that injection of the Anc80L65–SpCas9 system in vivo promotes hair cell survival in neomycin-treated mice.

### The delivery of Anc80L65–SpCas9 system to the inner ear ameliorates hearing loss induced by neomycin

To study the effect of the Anc80L65–SpCas9 system on hearing function in neomycin-treated mice, we measured auditory brainstem responses (ABRs), which represent the sound-evoked neural output of the cochlea. We observed severe hearing loss in the non-injected ears of neomycin-treated mice at P31, with ABR thresholds of 70–90 dB at 32 kHz compared to 25–65 dB in mice not treated with neomycin. In the Anc80L65–SpCas9–*Htra2* gRNA-injected ears of neomycin-treated mice, the ABR thresholds were significantly decreased at 8, 16, 24, and 32 kHz compared with the ears of neomycin-treated mice without injection of Anc80L65–SpCas9–*Htra2* gRNA, with average ABR thresholds 9–16 dB lower for the injected ears compared to the non-injected contralateral ears 2 weeks after the last dose of neomycin (Fig. 3e). There were no significant differences in ABR thresholds at 4 kHz between the two groups. Eight-kilohertz ABR waveforms from the single best injected ear and the representative non-injected ear are shown in Fig. 3f, with a 45 dB ABR threshold for the injected ear and 85 dB for the non-injected ear. At 4 weeks after the last dose of neomycin, the protective effect was sustained (Fig. 3g), with one best-performing mouse showing normal ABR thresholds at low frequencies (4 kHz: injected, 60 dB; non-injected, 75 dB; 8 kHz: injected, 35 dB; non-injected, 85 dB). ABR wave 1 amplitudes following 90 dB SPL stimulation at all the tested frequencies were greater in the injected ears than in the non-injected ears at 4 weeks after the last dose of neomycin (Fig. 3h, Additional file 1: Fig. S7A, Additional file 6). Correspondingly, the latencies of wave 1 stimulated by 90 dB SPL at all the tested frequencies were shorter in the injected ears compared to the non-injected ears (Fig. 3i, Additional file 1: Fig. S7B, Additional file 6). In one cohort of nine neomycin-treated mice, the protective effect of the Anc80L65–SpCas9 therapeutic system in vivo was sustained up to 8 weeks after the last dose of neomycin (Additional file 1: Fig. S8). The ABR thresholds of the injected ears of neomycin-treated mice were significantly decreased compared to the non-injected ears (8 kHz: injected, 68.6 ± 16.8 dB; non-injected, 88.6 ± 13.1 dB, *n* = 7, *p* < 0.05). There was no ABR threshold elevation in the injected ears (31.7 ± 4.1 dB at 8 kHz, *n* = 6) compared with the non-injected ears (32.9 ± 7.6 dB at 8 kHz, *n* = 7) in normal mice without neomycin exposure, which confirms that the Anc80L65–SpCas9 system does not affect the hearing function of wild-type mice (Additional file 1: Fig. S9A). The ABR thresholds of the injected ears in mice without neomycin treatment were still normal at 8 weeks after the injection of the Anc80L65–SpCas9 therapeutic system in vivo (Additional file 1: Fig. S9B).

### Targeting the Htra2 gene with the SaCas9 system

**Fig. 4:**
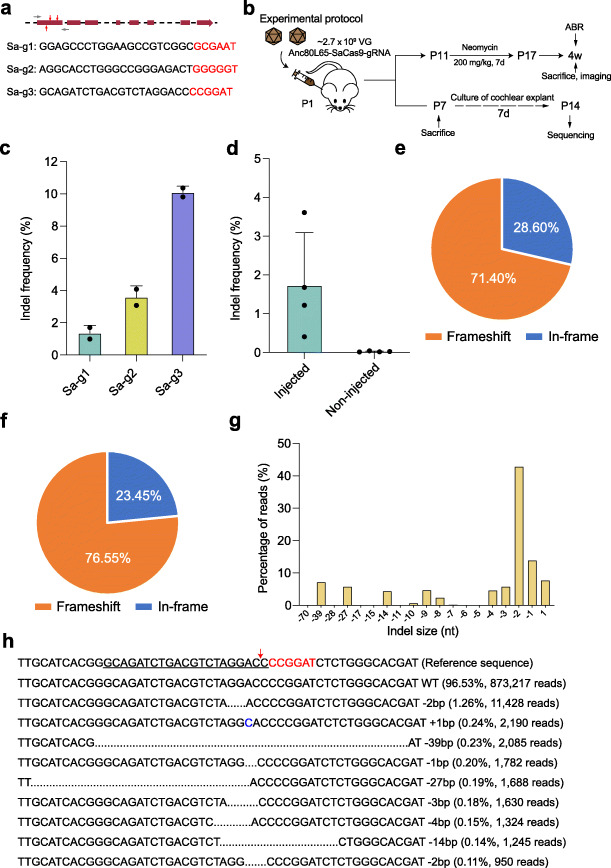
Targeting the *Htra2* gene with the SaCas9 system. **a** Design of the gRNAs. The three gRNA targeting sequences of the *Htra2* gene are listed. PAM sites are marked by red nucleotides. The red arrows indicate the exons targeted by gRNAs. The gray arrows indicate the primers. **b** Experimental overview of the in vivo studies of the Anc80L65–SaCas9 therapeutic system. **c** The indel frequency of the gRNAs was determined in HEI-OC1 cells by high-throughput sequencing. Dots represent individual values and bars represent the mean ± SD, *n* = 2. **d** The indel frequency of the gRNA (Sa-g3) from the injected and non-injected ears in vivo. Dots represent individual values and bars represent the mean ± SD, *n* = 4. **e** The indels causing in-frame versus frameshift mutations (percentages are shown) in the coding sequence after the plasmid of SaCas9–gRNA (Sa-g3) transfection in HEI-OC1 cells in vitro. **f** Indels causing in-frame versus frameshift mutations in the coding sequence after Anc80L65–SaCas9–*Htra2* gRNA injection in vivo. **g** Indel profiles from the cochleae injected with Anc80L65–SaCas9–*Htra2* gRNA in vivo. Negative numbers represent deletions and positive numbers represent insertions. **h** The top 10 indel mutations detected by deep sequencing after Anc80L65–SaCas9–*Htra2* gRNA injection in vivo

The Anc80L65–SpCas9 system was comprised of three AAV vectors (i.e., Anc80L65–SpCas9-N-terminal, Anc80L65–SpCas9-C-terminal, and Anc80L65–gRNA(g1-g2-g3)), which might have affected the transduction efficiency of the entire CRISPR/Cas9 system due to the limited injection volume of AAVs into the inner ear. Therefore, we designed a SaCas9 system to achieve a better protective effect using a single AAV vector. For the SaCas9 system, three gRNAs (Sa-g1, Sa-g2, and Sa-g3) were selected using the GPP sgRNA designer [50] (Fig. 4a). After comparing the indel frequencies of the three gRNAs in HEI-OC1 cells, Sa-g3 was chosen for subsequent experiments, and the SaCas9–gRNA was packaged into the Anc80L65 vector for the in vivo injection (Fig. 4b, c, Additional file 1: Fig. S10, Additional file 2). The average editing efficiency of the SaCas9 system at P14 was 1.73%, which was higher than that of the SpCas9 system under the same conditions (Fig. 4d, Additional file 2). The proportions of frameshift mutations with Sa-g3 were 71.40% and 76.55% in vitro and in vivo, respectively (Fig. 4e, f). The indel profiles showed that most CRISPR-induced variants were deletions, with the most common being 2-bp deletions (Fig. 4g, h). We also looked for off-target sites of the SaCas9 system, and no obvious indels were observed (Additional file 5).

### Effect of Anc80L65–SaCas9 system on hair cell survival and hearing function after neomycin exposure in vivo

**Fig. 5:**
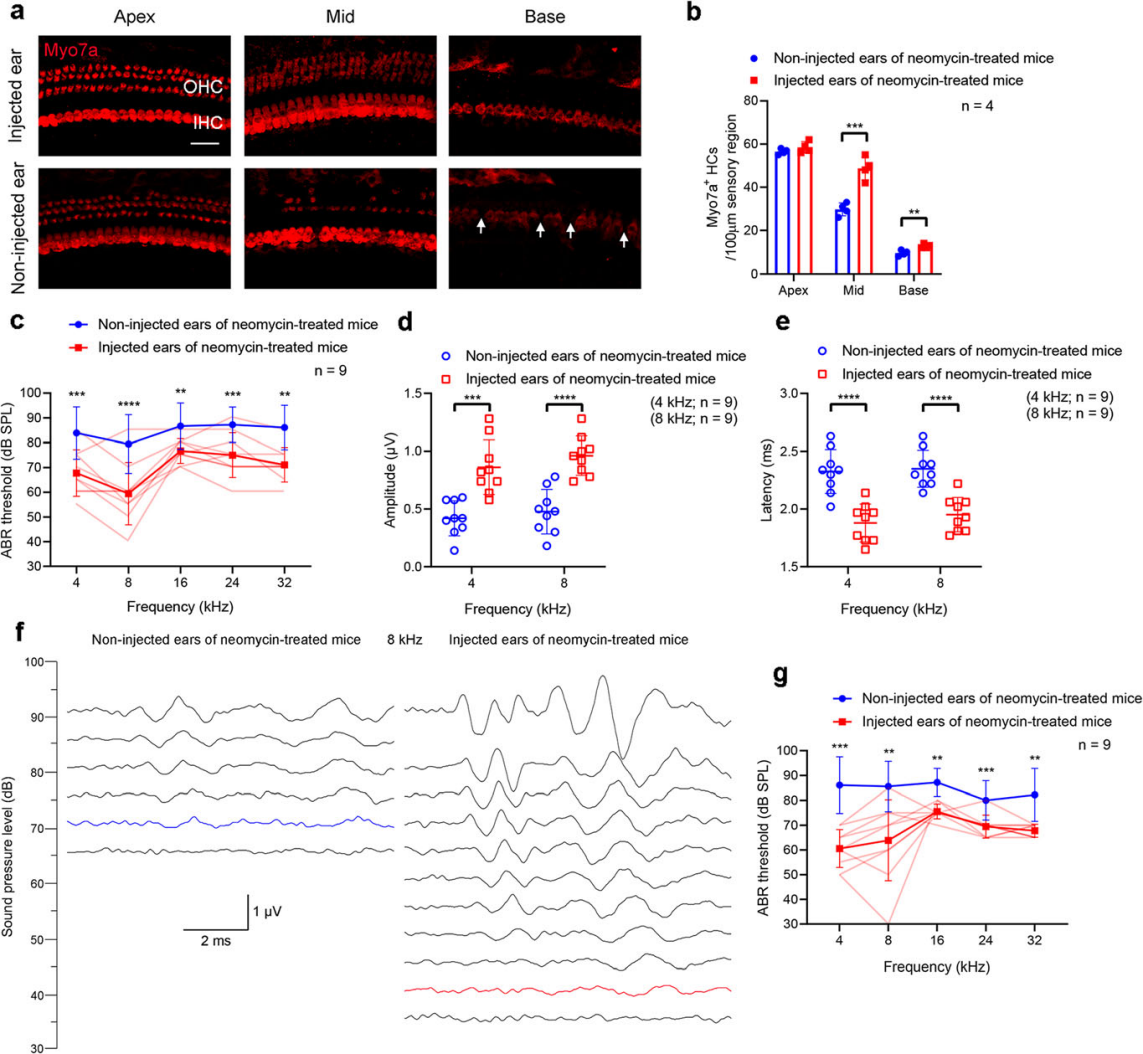
Anc80L65–SaCas9–*Htra2* gRNA injections protected hair cells and improved hearing function in neomycin-exposure mice. **a** Representative confocal microscopy images at 2 weeks after the last dose of neomycin from an injected cochlea and the contralateral (non-injected) cochlea. IHC inner hair cell, OHC outer hair cell. Arrows indicate the missing IHCs. Scale bar represents 20 μm long and applies to all images. **b** Quantification of the hair cells 2 weeks after the last dose of neomycin. Individual values are shown. Statistical tests in **b** are unpaired two-tailed Student’s *t*-tests. **c** ABR thresholds in injected and non-injected ears 2 weeks after the last dose of neomycin. Paired *t*-tests or Wilcoxon matched-pairs signed rank tests were used as applicable. **d**, **e** Peak amplitudes (**d**) and latencies (**e**) of ABR wave 1 evoked by 90 dB SPL at 4 and 8 kHz in the Anc80L65–SaCas9–*Htra2* gRNA-injected ears compared with the non-injected ears 2 weeks after the last dose of neomycin. Unpaired two-tailed Student’s *t*-tests were used. **f** 8-kHz ABR waveforms recorded at 2 weeks after the last dose of neomycin from the representative non-injected ear and the single best injected ear. The hearing thresholds are shown in blue and red for the non-injected ear and injected ear, respectively. The scale bar applies to all traces. **g** ABR thresholds in injected and non-injected ears 4 weeks after the last dose of neomycin. Paired *t*-tests or Wilcoxon matched-pairs signed rank tests were used as applicable. The lighter red lines in **c** and **g** represent the ABR thresholds of individual injected ears. **p < 0.01, ***p < 0.001, ****p < 0.0001

To determine the protective effect of the SaCas9 system in vivo, we delivered Anc80L65–SaCas9–*Htra2* gRNA (Sa-g3) into the inner ear through the scala media. The procedure for neomycin exposure was the same as for the SpCas9 system. The cochleae were dissected from the temporal bones after the ABR test, and immunohistochemical staining showed that there were more surviving hair cells in the middle and basal turns of the cochlea in the Anc80L65–SaCas9–*Htra2* gRNA-injected ears compared to the non-injected contralateral ears (Fig. 5a, b, Additional file 6). At 2 weeks after the last dose of neomycin, the ABR thresholds of the Anc80L65–SaCas9–*Htra2* gRNA-injected ears were significantly decreased at all the tested frequencies (4, 8, 16, 24, and 32 kHz) compared with the contralateral ears without injection of Anc80L65–SaCas9–*Htra2* gRNA (Fig. 5c). The average ABR thresholds were 10–20 dB lower for the injected ears compared to the non-injected contralateral ears in neomycin-exposed mice. We observed greater ABR wave 1 amplitudes and shorter latencies following 90 dB SPL stimulation at all the tested frequencies in the injected ears compared to the non-injected ears 2 weeks after the last dose of neomycin (Fig. 5d, e, Additional file 1: Fig. S11, Additional file 6). Eight-kilohertz ABR waveforms from the single best injected ear and the representative non-injected ear are shown in Fig. 5f, with a 40 dB ABR threshold for the injected ear and 70 dB for the non-injected ear. At 4 weeks after the last dose of neomycin, the protective effect of the Anc80L65–SaCas9 therapeutic system in vivo was still obvious (8 kHz: injected, 63.9 ± 16.4 dB; non-injected, 85.6 ± 10.1 dB, *n* = 9, *p* < 0.05), with the improvement of ABR threshold up to 50 dB at 8 kHz (Fig. 5g). The injection of Anc80L65–SaCas9–*Htra2* gRNA into the inner ear had no significant effect on the hearing function of wild-type ICR mice at 4 weeks (Additional file 1: Fig. S12) after the delivery of the AAV vectors.

We also measured distortion product otoacoustic emissions (DPOAEs) which represent the amplification provided by OHCs [51]. Two weeks after the last dose of neomycin, only 37.5% (3/8) and 12.5% (1/8) of the non-injected ears showed detectable DPOAE thresholds following 80 dB SPL stimulation at 4 kHz and 8 kHz, respectively, while all the injected ears showed detectable DPOAE thresholds at 4 kHz and 8 kHz (Additional file 1: Fig. S13). The DPOAE threshold of the Anc80L65–SaCas9–*Htra2* gRNA-injected ears was significantly decreased at 8 kHz compared with the non-injected ears (Additional file 6). The non-injected ears showed no detectable DPOAE thresholds following 80 dB SPL stimulation at middle and high frequency ranges (16–32 kHz), consistent with the severe OHC damage in the corresponding regions, while 37.5% (3/8) of the injected ears showed detectable DPOAE thresholds at 16 kHz, 24 kHz, and 32 kHz, respectively (Additional file 1: Fig. S13). Although the ABR thresholds of the injected ears at 16–32 kHz were decreased significantly compared to the non-injected ears (16 kHz, 24 kHz, and 32 kHz: injected, 76.7 ± 5.0 dB, 75.0 ± 9.0 dB, and 71.1 ± 7.0 dB, respectively; non-injected, 86.7 ± 9.4 dB, 87.2 ± 7.1 dB, and 86.1 ± 8.9 dB, respectively, *n* = 9, *p* < 0.05), the injected ears did not present conspicuous DPOAE recovery at the corresponding frequencies. We consider that the improvement of the ABR thresholds at 16–32 kHz in the injected ears may be mainly due to the protection of IHCs in the corresponding regions. Together, these results indicate that injection of Anc80L65–SaCas9–*Htra2* gRNA into the inner ears of neonatal mice partially rescues the hearing function after neomycin exposure in vivo.

## Discussion

Compared with hereditary deafness caused by genetic mutations, acquired hearing loss caused by noise, ototoxic drugs, infection, and aging is more common in the clinic. Previous studies demonstrated that aminoglycosides promote the formation of reactive oxygen species (ROS) that in turn induce apoptotic-like cell death via inhibition of the biosynthesis of mitochondrial proteins [5, 6, 16]. Numerous antioxidants and ROS scavengers have been used in clinical trials to attenuate ototoxicity [52] due to the key role of ROS in aminoglycoside-induced ototoxicity, and interfering with cell death signaling pathways promotes acute hair cell survival and attenuates drug-induced hearing loss following chronic aminoglycoside dosing [53]. However, the therapeutic effect of these strategies was not completely satisfactory. Omi/HtrA2 has been proposed to enhance caspase activation via multiple pathways other than cytosolic translocation [54], and Blink et al. [55] reported that Omi/HtrA2 can induce apoptosis without mitochondrial release. Therefore, we hypothesized that targeting the *Htra2* gene might protect hair cells from the ototoxicity caused by aminoglycosides.

There have been some successful studies showing hearing improvement through the traditional strategy of gene overexpression in mouse models with gene defects of *Ush1c*, *Otof*, *Tmc1*, *Vglut3*, and *Kcnq1* [56–62]. Transfer of the therapeutic wild-type genes offers a potential treatment strategy for recessive genetic diseases, but this approach may require repeated administration to obtain a lifetime therapeutic effect, which increases the risk of inducing side effects, including clinically relevant immunogenicity [63]. The CRISPR/Cas9 technology can specifically edit target genes and offers promising alternatives for diseases such as Duchenne muscular dystrophy [64], metabolopathies [65], and deafness [33], providing a potential one-time treatment strategy for genetic diseases. Theoretically, after editing the pathogenic genes, the prevention or treatment of disease can be achieved permanently compared to the strategies of overexpression or RNA interference. Using the CRISPR/Cas9 technology, Bence et al. [34] selectively and efficiently disrupted the mutant *Tmc1* allele in *Beethoven* mice, and this prevented deafness in *Beethoven* mice up to 1 year post-injection.

In the current study, we used two CRISPR/Cas9 systems, SpCas9 and SaCas9, to knock out the *Htra2* gene, and both systems reached the goal of hearing protection to some extent. It is exceedingly difficult to package SpCas9 into a single AAV2/Anc80L65 vector due to its limited packing capacity, but we successfully constructed Anc80L65–SpCas9 system with the split-SpCas9 scheme in the field of gene therapy for hearing loss. However, the delivery of the SpCas9 system using three AAV vectors might have affected the transduction efficiency of the whole therapeutic system in hair cells, which may be one of the factors leading to lower editing efficiency compared with the SaCas9 system. Moreover, the different promoters and viral doses used for the SpCas9 and SaCas9 system might be other key confounders leading to different therapeutic effects between the two systems.

The CRISPR/Cas9 technology was used to knock out the *Htra2* gene in the inner ear of mice both in vitro and in vivo, and we observed a protective effect of the CRISPR/Cas9–*Htra2* system on cochlear hair cells against neomycin-induced ototoxicity. The injected ears showed 10 to 30 dB improvement of ABR thresholds in neomycin-treated mice compared with the non-injected ears at 8 kHz for the SaCas9 system. Aminoglycosides are one of the most common causes of acquired SNHL in the clinic and usually lead to profound deafness. A similar corresponding improvement of ABR thresholds would help patients with extensive aminoglycoside-induced hearing loss detect high-decibel sound in the environment and avoid the surgery of cochlear implantation. These patients could acquire better auditory experience with hearing aids.

Although the auditory function was improved significantly, the ABR thresholds did not recover to normal, especially in the high frequencies. We conjecture that the protection of hair cells from the whole cochlea with AAV–CRISPR/SpCas9 system in vitro was due to high transduction efficiency of Anc80L65 in IHCs and OHCs. A satisfactory viral dose can be achieved in in vitro culture system while the viral dose will decrease sharply on account of the limited injection volume of virus into the inner ear. A previous study demonstrates that the volume of the endolymphatic space of adult mice is approximately 0.78 μl [66]. Our data demonstrate that Anc80L65-EGFP transduced 100% of the IHCs and about 90% of the OHCs with a viral dose of 5 × 10^9^ VG after in vivo injection. However, for the therapeutic system, the actual viral dose per cochlea for Anc80L65–SpCas9, Anc80L65–*Htra2* gRNA, and Anc80L65–SaCas9–*Htra2* gRNA was 1.8 × 10^9^ VG, 0.9 × 10^9^ VG, and 2.7 × 10^9^ VG, respectively. The lower viral dose will result in lower transduction efficiency in OHCs [67]. In the in vivo experiment, no obvious protective effect was observed in OHCs of the basal turn of the cochlea, which is consistent with the lower transduction efficiency of Anc80L65 in OHCs [68, 69] and the susceptibility of basal-turn OHCs to aminoglycoside-induced ototoxicity. These data suggest that there is an urgent need for improved transduction efficiency of viral vectors in cochlear OHCs and advanced production technique aimed at yielding high-titer virus suitable for clinical use. In addition, improvement in editing efficiency, which could generate better protective effect, is another critical issue to be addressed. With the development of genome-editing strategy, it is hopeful that enhanced editing efficiency would be achieved in mammalian non-proliferating cells in the future.

In this study, no mice developed any behavioral signs of vestibular damage after neomycin exposure in vivo. We assume that the neomycin damage pattern used in our study was insufficient to cause vestibular dysfunction in mice. At P11, mice received a daily subcutaneous injection of neomycin (200 mg/kg) continuously for 7 days. Within the observation period, neomycin-exposed mice showed no obvious symptoms of postural asymmetries, including head deviation, trunk curvature, forelimb extension, circling compulsory movements, and head nystagmus. Moreover, Tsuji et al. [70] performed a quantitative assessment of vestibular hair cells and Scarpa’s ganglion cells in temporal bones from patients who had aminoglycoside ototoxicity (streptomycin, kanamycin, and neomycin). For neomycin, the data in their study suggested that there was little hair cell ototoxic effect in the vestibular sense organs. However, it was reported that the severity of vestibular damage was in the order of streptomycin, gentamicin, amikacin, and netilmicin in Guinea pigs [71]. Therefore, it is meaningful to investigate whether the AAV–CRISPR/Cas9 strategy protects the vestibular system from aminoglycoside-induced ototoxicity in the future work.

The protective effect of the AAV–CRISPR/Cas9 system in vivo was sustained up to 8 weeks after neomycin exposure, and this system was proved to be safe for the auditory function of wild-type mice within the observation period. However, to realize the clinical translation, there is a need for evaluating longer-term safety of this AAV–CRISPR/Cas9 strategy and identifying the therapeutic time-window in adult animals.

Omi/HtrA2 is a proapoptotic mitochondrial serine protease that is released into the cytoplasm following apoptotic insult [72]. Wang et al. observed that increased expression of Omi/HtrA2 in aging rats augmented myocardial ischemia/reperfusion injury by stimulating myocardial apoptosis [73], which suggests that strategies to inhibit Omi/HtrA2 may protect against heart injury. However, a neurodegenerative phenotype with parkinsonian features has been defined in Omi/HtrA2 knockout mice [74], and Strauss et al. performed a mutation screening of the *Omi/HtrA2* gene in German Parkinson’s disease (PD) patients and identified a heterozygous G399S mutation in four (4/518) patients [75], which indicates that loss of function of Omi/HtrA2 in the central nervous system (CNS) may be linked to PD. The pathological hallmark of PD is depigmentation of the substantia nigra and locus coeruleus with neuronal loss in the pars compacta of the substantia nigra [76]. Landegger et al. has demonstrated that Anc80L65-eGFP injected via round window membrane transduced Purkinje neurons in the cerebellum [38] which is not the affected brain area in PD. In our work, the AAV–CRISPR/Cas9 system was injected into the scala media of the cochlea. As safety assessment is a particularly important aspect of gene therapy, it is necessary to examine whether the therapeutic system enters into the CNS and which encephalic regions and types of neurons are transduced with such injection route in future study. Together, to take a further step towards clinical translation of this CRISPR/Cas9-based strategy, it requires further investigation to evaluate the potential influence of this treatment on the CNS.

## Conclusions

In conclusion, this work provides compelling evidence that AAV-mediated delivery of the CRISPR/Cas9–*Htra2* system into the inner ear prevents aminoglycoside-induced hearing loss. This outcome is noteworthy because it demonstrates the feasibility of CRISPR/Cas9-mediated disruption of an apoptosis-related gene to prevent ototoxic deafness. Given that most acquired non-inherited SNHL is related to apoptosis, CRISPR/Cas9-based strategies would be preventative options for this type of hearing loss.

## Methods

### Experimental design

This study was designed to investigate the possibility of preventing ototoxic deafness, one of the most common types of acquired non-inherited SNHL, via disrupting an apoptosis-related gene with the CRISPR/Cas9 technology. We selected *Htra2* as the target gene and developed two CRISPR/Cas9 systems, SpCas9 and SaCas9. The indel frequency of each gRNA for the SpCas9 or SaCas9 system was measured in HEI-OC1 cells by HTS. AAV2/Anc80L65 was used to deliver CRISPR/Cas9 systems in vitro and in vivo. To identify whether disrupting the *Htra2* gene could protect cochlear hair cells against neomycin-induced ototoxicity, we performed the in vitro experiment in the cochlear explants firstly, measuring the hair cell survival and apoptosis by immunohistochemistry and confocal microscopy. The AAV–CRISPR/Cas9 systems were injected into the inner ear of wild-type mice at P1, and the editing efficiency in vivo was evaluated by HTS. The protective effect in vivo was evaluated using immunohistochemical analysis and measurement of ABR thresholds, peak amplitudes, and latencies of ABR wave 1. Non-injected ears served as controls. Each experiment was replicated as indicated by *n* values in the figures or tables. All the experimental samples were included in the analysis, with no data excluded. Ears of both sides were randomly assigned to injected and non-injected groups, and wild-type mice were randomized into neomycin-treated and neomycin-untreated experiments without regard to gender. Investigators were not blinded when performing cell counting in each experiment. ABRs were obtained by investigators blinded to the experimental conditions. Sample size was selected on the basis of statistical significance. A power analysis was not performed.

### Animals

All animals were bred and housed in the Department of Laboratory Animal Science of Fudan University. All animal experiments were approved by the Institutional Animal Care and Use Committee of Fudan University, and we adhered to the NIH Guide for the Care and Use of Laboratory Animals. We used C57BL/6J wild-type mice for the in vitro experiments and Institute of Cancer Research (ICR) wild-type mice for the in vivo experiments due to the high fertility of ICR mice.

### Construction of the Cas9 and sgRNA expression plasmids

We designed two CRISPR/Cas9 systems to target genomic sites in *Htra2*. The SpCas9 system had two different combinations. The first SpCas9 system included three different plasmids with SpCas9, one of the three sgRNAs and GFP constructed in one plasmid, while the second system consisted of the SpCas9 plasmid and a single plasmid containing all three sgRNAs concatenated together in a series. The sequences of the sgRNAs for the SpCas9 system were GCC TAG CGG GGA TAC TTA TG (Sp-g1), CTG CAG AAC ACG ATC ACA TC (Sp-g2), and CGC GAT GCA GAA ACT CCC TA (Sp-g3). The SaCas9 system was constructed with SaCas9 and sgRNA in one plasmid, and we constructed three SaCas9–sgRNA plasmids with three different sgRNA sequences GGA GCC CTG GAA GCC GTC GGC (Sa-g1), AGG CAC CTG GGC CGG GAG ACT (Sa-g2), and GCA GAT CTG ACG TCT AGG ACC (Sa-g3).

### Cell culture and transfection

The HEI-OC1 cell line was maintained in Dulbecco’s modified Eagle medium (DMEM) (Gibco, #11965092) supplemented with 5% FBS (Gibco, #16140071), 100 U/ml penicillin, and 100 mg/ml streptomycin (penicillin/streptomycin, Millipore, #516106) at 37 °C and 5% CO_2_. For transfection, HEI-OC1 cells were plated onto 12-well plates, and different combinations of plasmids were mixed with Lipofectamine 2000 (Life Technologies, #11668019) in Opti-MEM (Life Technologies, #31985062) according to the manufacturer’s instructions. A total of 750 ng of SpCas9 plasmid was co-transfected with 250 ng of sgRNA expression plasmid. GFP-positive cells were sorted by FACS at 48 h post-transfection and then cultured for 7 days.

### Mouse genomic DNA isolation and PCR

For the in vitro experiments, genomic DNA was isolated from HEI-OC1 cells after transfection with the CRISPR/Cas9 systems, and genomic DNA from cultured cochlear tissues was isolated 8 days after transduction with the Anc80L65–SpCas9 system using the QuickExtract DNA Extraction Solution (Epicentre, #QE09050) following the manufacturer’s instructions. For the in vivo experiments, genomic DNA was extracted from the whole cochlear tissue of the inner ear that was injected with the SpCas9 or SaCas9 system at P1, dissected at P7, and cultured for another 7 days in vitro. GFP-positive cells were sorted by FACS. Genomic DNA from non-transfected HEI-OC1 cells, non-transduced cultured cochlear tissue, and non-injected cochlear tissue were used as controls for each experiment. The target sites were PCR-amplified by nested PCR amplification and purified with a Gel DNA Extraction Kit (Vazyme, # DC301) for deep sequencing.

### Western blot analysis

Proteins extracted from HEI-OC1 cells with Radio-Immunoprecipitation Assay (RIPA) buffer (Protein Biotechnology, #PP109) (including Protease Inhibitor Cocktail, Roche, #11697498001) were electrophoresed in 10% Bis-Tris protein gels and transferred to PVDF membranes (GE Healthcare Life Sciences, #10600023). After being blocked in 5% non-fat dry milk (Cell Signaling Technology, #9999S), blots were probed with primary antibodies at 4 °C overnight: rabbit anti-HtrA2/Omi (1:1000, Cell Signaling Technology, #9745) and mouse anti-β-actin (1:1000, Cell Signaling Technology, #8457s). The membranes were then incubated with the HRP-conjugated secondary antibodies (1:5000) for 1 h at room temperature. Signals were detected with Meilunbio® fg super sensitive ECL luminescence reagent (Meilunbio, #MA0186).

### Off-target analysis

The potential off-target sites were predicted by CasOFFinder software, and specific primers were designed. Specifically, edited genomic DNA was used as the template to amplify the off-target genomic sites by PCR using flanking HTS primer pairs as specified in Additional file 7. PCR products were purified with a QIAquick PCR Purification Kit (Qiagen, #28104) and sequenced on a MiSeq high-throughput DNA sequencer (Illumina).

### Viral vector preparation

Anc80L65 capsids were used to package the SpCas9 and SaCas9 systems. All the plasmids were sequenced before packaging into AAV2/Anc80L65. These viral vectors were produced by the Biolink Company (Shanghai, China). The original titers of AAV2/Anc80L65–CAG–SpCas9–WPRE–polyA, AAV2/Anc80L65–U6–(*Htra2*-gRNA) × 3–CAG–eGFP (Super)–bGHpolyA, and AAV2/Anc80L65–CMV–SaCas9–bGHpolyA–U6–(*Htra2*-gRNA) were 5.37 × 10^12^ viral genomes (VG)/ml, 5.51 × 10^12^ VG/ml, and 5.38 × 10^12^ VG/ml, respectively, as determined by real-time qPCR.

### Cochlear explant culture, application of viral vectors, and the in vitro neomycin damage model

The cochleae of wild-type postnatal day 1 (P1) C57BL/6J mice were dissected, and the organ of Corti was cultured in DMEM/F12 (Gibco, #11330032) with 1% N2 supplement (Gibco, #17502048), 2% B27 supplement (Gibco, #17504044), and 50 mg/ml ampicillin (Sigma, #A5354) at 37 °C with 5% CO_2_. For the viral transduction, Anc80L65-EGFP was applied to the cochlear explants 2 h after culture, and the explants were incubated in the nutrient solution with viral vectors for 48 h. The working titer of Anc80L65-EGFP was 2 × 10^11^ VG/ml. After another 6 days of culture in a DMEM/F12 mixture without virus, the cochlear explants were harvested for immunohistochemical staining. To study the protective effect in vitro, the viral vectors of the CRISPR/Cas9 system (SpCas9) were applied to the cochlear explants 2 h after culture, and the explants for the AAV-treatment groups were incubated in the nutrient solution with AAV vectors for 48 h. The working titers of Anc80L65–SpCas9 and Anc80L65–*Htra2* gRNA were 2 × 10^11^ VG/ml and 1 × 10^11^ VG/ml, respectively. The medium was then changed to a DMEM/F12 mixture without virus, and the explants were cultured for another 6 days. After total 8 days of culture (2 days with virus and 6 days without virus), neomycin (1 mM, Sigma, #N6386) was applied, and the cochlear explants were cultured in DMEM/F12 mixed with neomycin for 6 h. After 24 h of recovery in DMEM/F12 medium without neomycin, the cochlear explants were harvested for immunohistochemical staining.

### Microinjection of AAV vectors into the inner ear and the in vivo neomycin damage model

The AAV vectors were injected into the inner ear of wild-type ICR mice. P1 mice were anesthetized with low temperature exposure by placing them on ice, and a skin incision was made behind the right ear to expose the otic bulla and the stapedial artery. For the in vivo viral transduction experiment, Anc80L65-EGFP was injected into the scala media of the cochlea [77] with a total volume of 0.5 μl per cochlea, and the cochleae were dissected and fixed for immunohistochemical staining 10 days after injection. After mixing Anc80L65–SpCas9 and Anc80L65–(*Htra2*-gRNA) × 3–eGFP at a 2:1 ratio, a total volume of 0.5 μl per cochlea was injected into the scala media of the cochlea to test its protective effect in vivo. The injection volume of Anc80L65–SaCas9–(*Htra2*-gRNA) was also 0.5 μl per cochlea. Glass micropipettes (WPI, Sarasota, FL) held by a Nanoliter Microinjection System (WPI) were used to deliver the AAV vectors, and the speed of injection was controlled by a micromanipulator at 3 nl/s [67]. After injection, the incision was closed with sutures and the pups were placed on a 42 °C heating pad for recovery. The pups were returned to their mother after fully recovering within 10 min. Standard post-operative care was applied after surgery. The injected mice were sacrificed at different ages depending on the experiment. At P11, which was the tenth day after being injected with the viral vectors, mice received a daily subcutaneous injection of neomycin (200 mg/kg) continuously for 7 days.

### Auditory testing

ABR and DPOAE measurements were recorded in a soundproof chamber using the RZ6 Acoustic System (Tucker-Davis Technologies, Alachua, FL, USA) at 2 weeks, 4 weeks, and 8 weeks after the last dose of neomycin as previously described [25]. In brief, mice of either sex were anesthetized by intraperitoneal injection of xylazine (10 mg/kg) and ketamine (100 mg/kg), and needle electrodes were inserted into the subcutaneous tissues of the vertex (the reference electrode), the mastoid portion (the recording electrode), and the rump of the animal (the ground electrode). Each animal was stimulated by 5-ms tone pips, and ABR potentials were evoked and subsequently amplified (10,000 times), filtered (300 Hz–3 kHz passband), and averaged (1024 responses) at each sound pressure level (SPL). Tone burst sound stimuli were presented at 4, 8, 16, 24, and 32 kHz to test the frequency-specific hearing thresholds as previously described [78]. The threshold of a certain frequency was determined as the lowest SPL at which any ABR wave could be detected upon visual inspection. The amplitude of wave 1 of the ABR was measured from the peak of wave 1 to the following trough [79].

For DPOAEs [34], the f1 and f2 primary tones (f2/f1 = 1.2) were presented with f2 at 4, 8, 16, 24, and 32 kHz and L1 − L2 = 10 dB sound pressure level (dB SPL). For each f2/f1 primary pair, L2 was swept in 10-dB increments from 20 to 80 dB SPL. Waveform and spectral averaging were used at each level to increase the signal-to-noise ratio of the recorded ear-canal sound pressure. DPOAE threshold was defined from the average spectra as the f2-level producing a DPOAE of magnitude 5 dB SPL above the noise floor. The mean noise floor level was under 0 dB across all frequencies.

During the whole procedure of acoustic testing, the mice were placed on a heating pad covered by a sterile drape to maintain their body temperature. The animals were returned to the breeding center after they recovered from anesthesia and regained voluntary movement. In general, thresholds were defined by two independent observers. Data were analyzed and plotted using GraphPad Prism 8, and threshold mean ± standard deviation (SD) are presented unless otherwise stated.

### Immunohistochemistry and confocal microscopy

For the in vitro experiments, cochlear explants were fixed with 4% paraformaldehyde for 60 min at room temperature and preserved in PBS until staining. For the in vivo experiments, the cochleae were dissected from the temporal bones at different ages after the injection of viral vectors. The round and oval window membranes of the cochleae were opened, and the inner ears were flushed with 4% paraformaldehyde. Then, the cochlear samples were fixed with 4% paraformaldehyde for 60 min at room temperature. After rinsing with PBS three times, the cochleae were soaked in 10% EDTA for 3 days for decalcification. The entire sensory epithelium was divided into three pieces of equal length designated the basal, middle, and apical turns of the cochlea and preserved in PBS until staining. All samples for the in vitro and in vivo experiments were permeabilized and blocked in PBS containing 1% Triton X-100 (1% PBS-T) and 10% donkey serum for 1 h at room temperature. All primary and secondary antibodies were diluted in 1% PBS-T. To observe the eGFP expression in the hair cells of the cochlear sensory epithelium after the application of AAV2/Anc80L65-EGFP, we used rabbit anti-Myo7a (1:500 dilution, Proteus BioSciences, #25-6790) and chicken anti-GFP (1:1000 dilution, Abcam, #ab13970) primary antibodies to label hair cells and eGFP, respectively. For measuring apoptosis in the cochlear sensory epithelium, rabbit anti-cleaved caspase3 (1:1000 dilution, Cell Signaling Technology, #9664S) and mouse anti-Myo7a IgG_2a_ (1:500 dilution, Santa Cruz Biotechnology, #sc-74516) primary antibodies were used to detect the expression of caspase3 in hair cells. To determine the expression of HTRA2 in hair cells of the cochlear sensory epithelium, rabbit anti-HTRA2 (1:250 dilution, Sigma-Aldrich, #SAB4501221) and mouse anti-Myo7a IgG_2a_ (1:500 dilution, Santa Cruz Biotechnology, #sc-74516) primary antibodies were used. Omission of the primary antibody served as the negative control. Appropriate Alexa-conjugated secondary antibodies were used for detection, and DAPI was used to label the nuclei (1:1000 dilution, Sigma, #D9542). The fluorescent Z-stack images were collected using a Leica TCS SP8 laser scanning confocal microscope and a × 40 objective. The scanning parameters of the confocal microscope, including laser power, smart gain, and smart offset, were kept identical across different samples which were stained with the same antibodies and under the same staining conditions. The maximum intensity projections of optical confocal sections are shown in the figures.

### Cell counting

For cell counting, the numbers of Myo7a^+^ HCs, eGFP^+^/Myo7a^+^ cells, caspase3^+^ cells of the sensory epithelium, and caspase3^+^/Myo7a^+^ cells were counted in every 100-μm region of the apical, middle, and basal turns of the cochlea using ImageJ software. For the in vitro experiments, at least four 100-μm regions from at least four independent cochleae were counted to derive an average value for each “Apex,” “Mid,” and “Base” region. For the in vivo experiments, four samples in each group from four independent experiments were collected. The transduction efficiencies of AAV vectors targeting hair cells were calculated as the ratio of eGFP^+^/Myo7a^+^ cells to Myo7a^+^ cells. The results are presented as the mean ± SD.

### Statistical analysis

Statistical analyses were performed with GraphPad Prism 8. For comparison of two independent samples, we applied different methods of statistical analysis for different conditions. In the case of abnormal distribution, a non-parametric statistical test (Mann–Whitney *U*-test) was used to analyze the data. An unpaired *t*-test with Welch’s correction was performed if the data conformed to a normal distribution but equal variance could not be assumed. Under the condition of normal distribution and equal variance, an unpaired two-tailed Student’s *t*-test was used to determine statistical significance. When comparing the ABR thresholds between the non-injected and injected ears of neomycin-exposed mice, paired *t*-tests were applied in the case of normal distribution of the difference between two groups of data, while Wilcoxon matched-pairs signed-rank tests were used if the difference between two groups of data did not follow a normal distribution. *P* < 0.05 was considered statistically significant. Mean ± SD, statistical analysis, and *P* values for each experiment were presented in detail (see Additional file 6).

## Supplementary Information

**Additional file 1: Figure S1–S13** with figure legends.

**Additional file 2.** The results of HTS.

**Additional file 3.** The indel profiles from the three sgRNAs of SpCas9 system in HEI-OC1 cells.

**Additional file 4.** Source data of blots and gels.

**Additional file 5.** The indel frequency of the off-target sites.

**Additional file 6. **Statistical analysis and *P* values for each experiment.

**Additional file 7.** The list of primer sequence.

**Additional file 8.** Review history.

## Data Availability

The datasets supporting the conclusions of this article are included within the article and its additional files. AAV and CRISPR/Cas9 constructs are available from Addgene. The original data of HTS, including on-target and off-target results, have been submitted to the NCBI Sequence Read Archive under accession number PRJNA674478 [80].
