# Supplementary material for: Prevention of acquired sensorineural hearing loss in mice by in vivo Htra2 gene editing
Source: Genome Biol. 2021 Mar 22;22:86. doi: 10.1186/s13059-021-02311-4 (PMC7983387; doi:10.1186/s13059-021-02311-4)
Supplement: Supplementary file 1 — Additional file 1: Figure S1–S13 with figure legends. [file 13059_2021_2311_MOESM1_ESM.pdf]

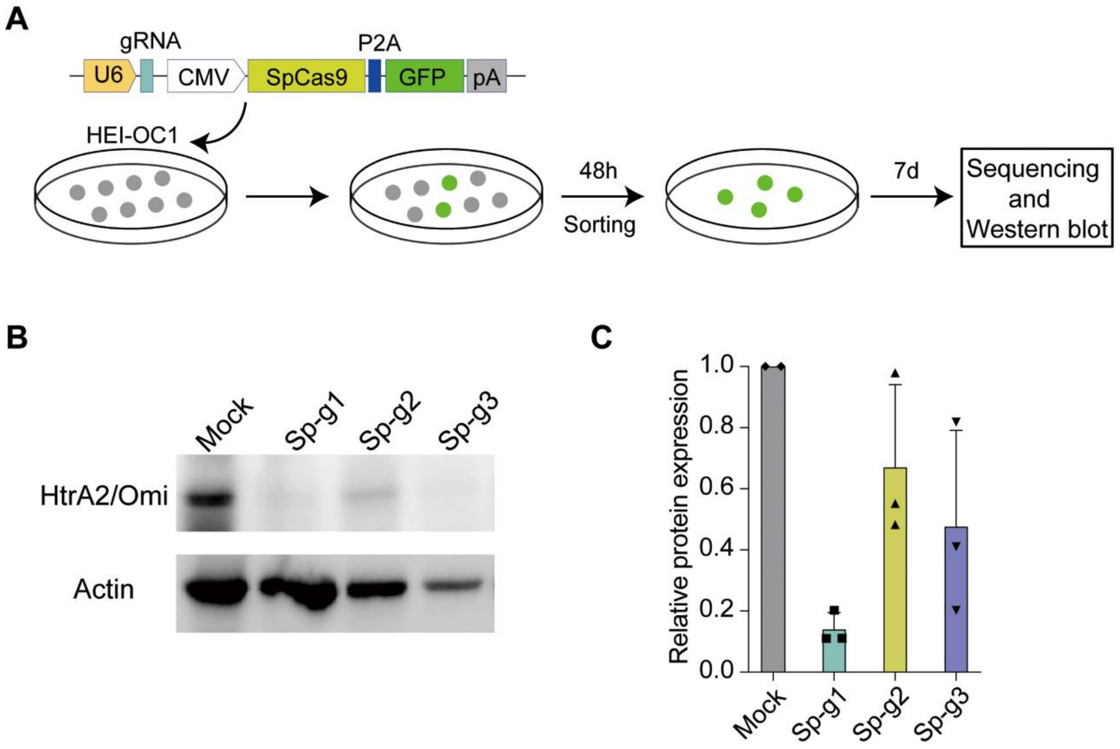


**Fig. S1. *In vitro* genome editing of the *Htra2* gene with the SpCas9 therapeutic system.** (**A**) Experimental procedure for the *in vitro* detection of genome editing. The plasmid contained SpCas9, gRNA, and GFP. (**B**–**C**) The western blot results of protein disruption after transfection with Sp-g1, Sp-g2, and Sp-g3, respectively, in HEI-OC1 cells. The statistical data were obtained from three independent repeated experiments. Dots represent individual values, and bars represent the mean ± SD.

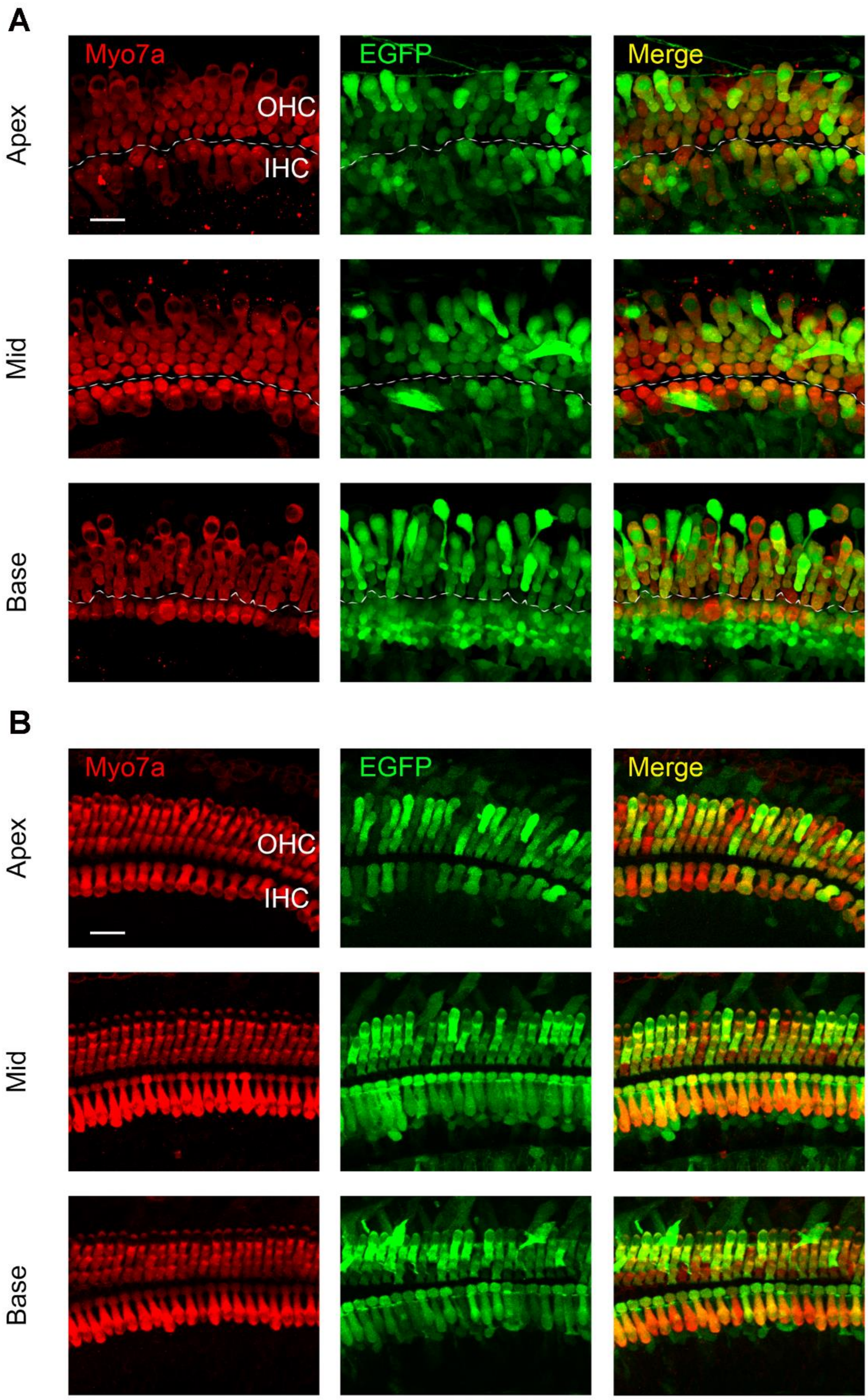


**Fig. S2. Transduction of AAV2/Anc80L65-CMV-EGFP in hair cells of the cochlea *in vitro* and *in vivo*.** (**A**) Transduction of AAV2/Anc80L65-CMV-EGFP in hair cells of

the cochlea at a dose of $2 \times 10^{10}$ VG *in vitro*. The confocal images indicate high transduction efficiencies of the virus in inner and outer hair cells (IHCs and OHCs) after being cultured for 7 days *in vitro*. The dashed curve represents the boundary between IHCs and OHCs. (**B**) Transduction of AAV2/Anc80L65-CMV-EGFP in hair cells of the cochlea at a dose of $5 \times 10^{9}$ VG *in vivo*. The cochleae were dissected for immunostaining 10 days after injection. After *in vivo* injection into the scala media of the cochlea, AAV2/Anc80L65-EGFP transduced 100% of the IHCs and about 90% of the OHCs in the whole cochlea. IHC: inner hair cell, OHC: outer hair cell. Both scale bars (in **A** and **B**) represent 20 μm long and apply to all images.

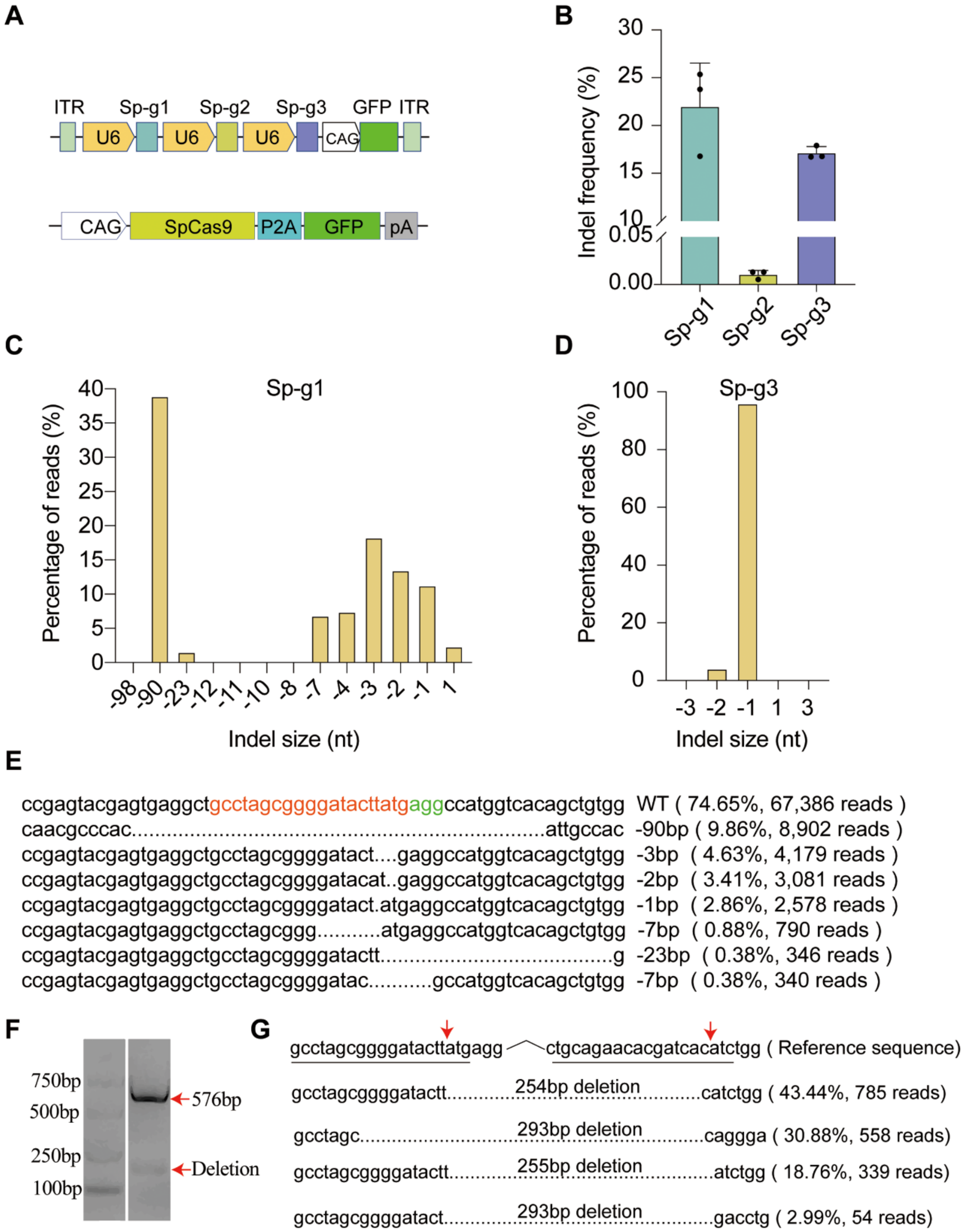


**Fig. S3. *In vitro* genome editing with the SpCas9 therapeutic system in which the three gRNAs were constructed in one plasmid.** (**A**) One plasmid contained three gRNAs and GFP, and the other contained SpCas9 and GFP. (**B**) The indel frequency of the three gRNAs was measured in HEI-OC1 cells by high-throughput sequencing. Dots

represent individual values and bars represent the mean ± SD, n = 3. (**C** and **D**) Indel profiles of Sp-g1 (**C**) and Sp-g3 (**D**) from HEI-OC1 cells co-transfected with two plasmids. Negative numbers represent deletions and positive numbers represent insertions. (**E**) The top 7 indel mutation types detected by deep sequencing after co-transfection of SpCas9 and gRNAs. (**F**) Results of agarose gel separation showing the deletions of large fragments between the target site of Sp-g1 and Sp-g2. (**G**) The deletion mutations between the target site of Sp-g1 and Sp-g2 detected by deep sequencing. nt: nucleotides, bp: base pairs.

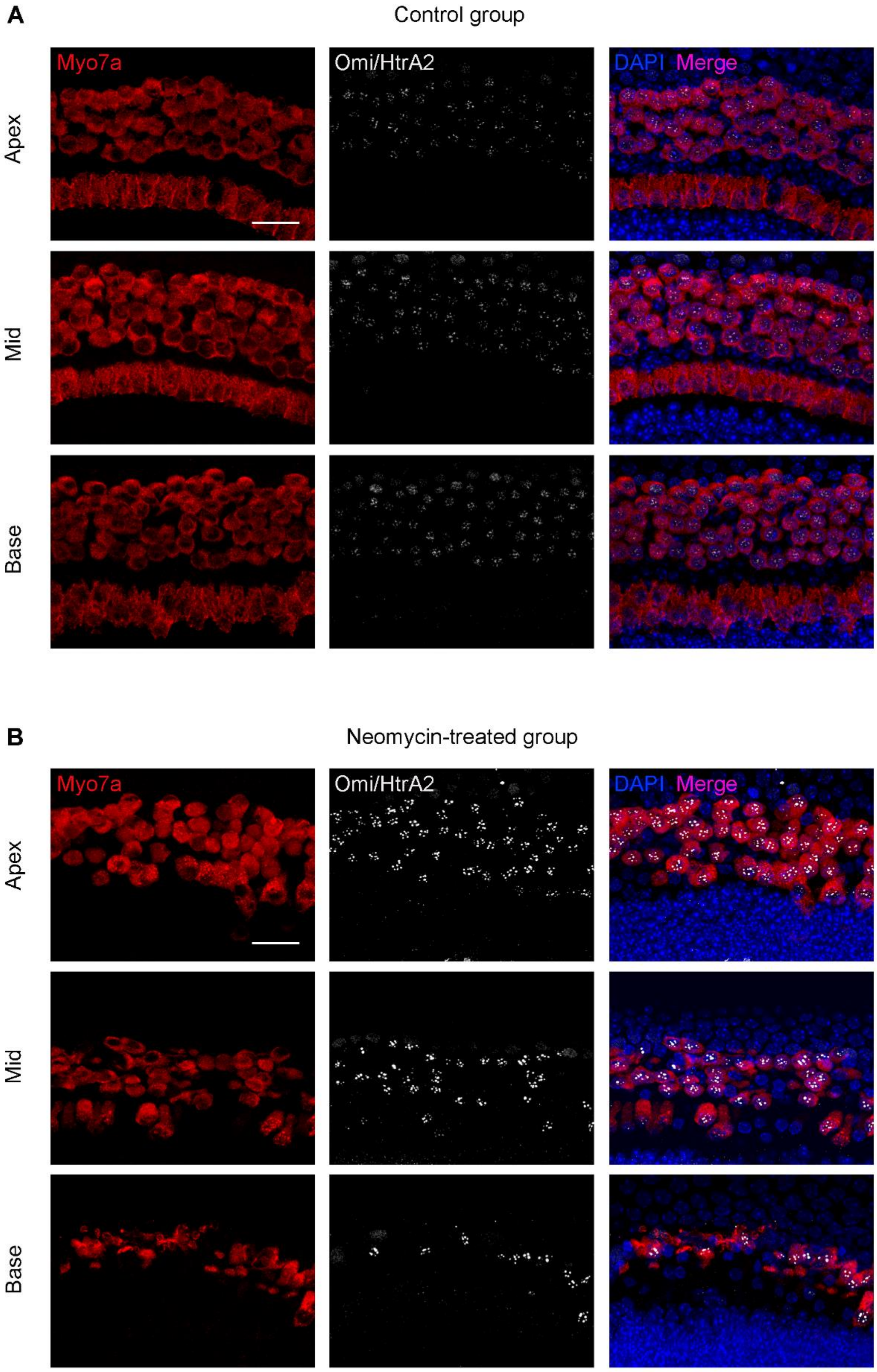


**Fig. S4. The difference in expression of Omi/HtrA2 protein in the control group**

**and the neomycin-treated group.** (**A**) Representative confocal images of the expression of the Omi/HtrA2 protein in the control group. The Omi/HtrA2 protein was expressed in the nucleus of hair cells in the normal explants. (**B**) Representative confocal images of the expression of the Omi/HtrA2 protein in the neomycin-treated group. The expression of the Omi/HtrA2 protein was increased after neomycin exposure. Both scale bars (in **A** and **B**) represent 20 µm long and apply to all images.

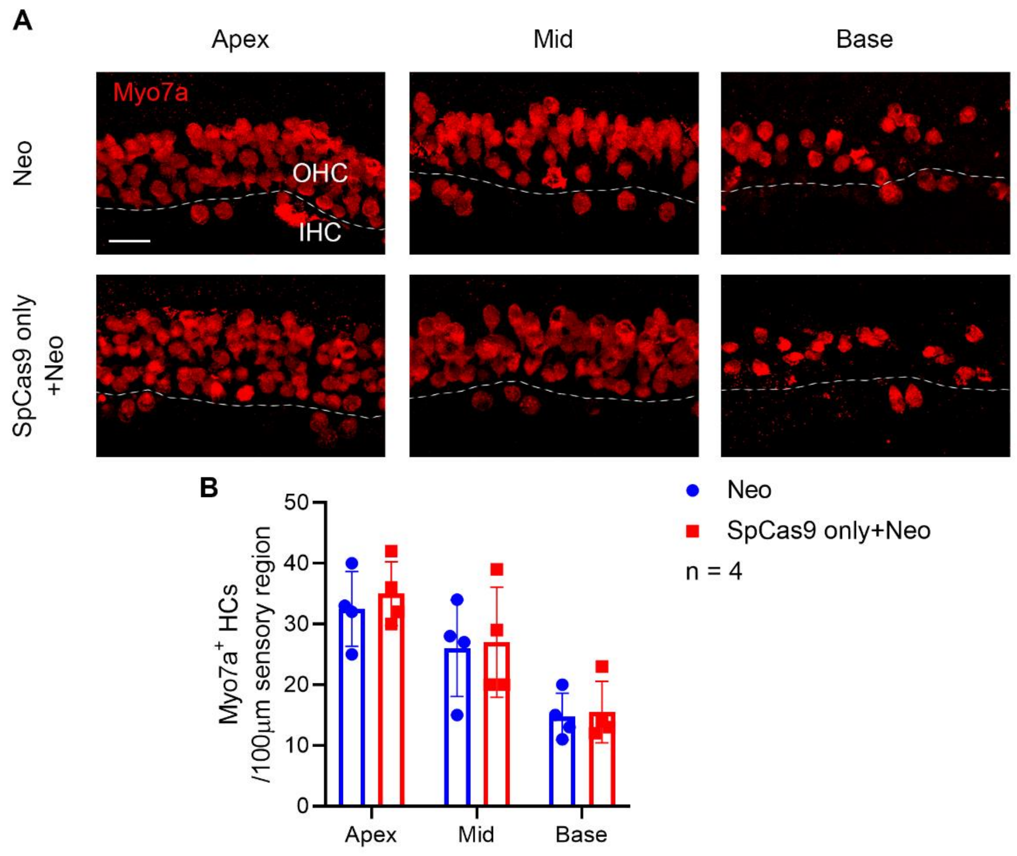


**Fig. S5. The application of Anc80L65–SpCas9 alone cannot protect hair cells from neomycin-induced injury *in vitro*.** (**A**) Representative confocal images of neomycin-treated cochlear explants with or without Anc80L65–SpCas9 application. IHC: inner hair cell, OHC: outer hair cell. Scale bar represents 20 μm long and applies to all images. (**B**) Comparison of hair cells between the neomycin-treated groups with or without Anc80L65–SpCas9 application. The numbers of Myo7a$^{+}$ hair cells of the apical, middle, and basal turns of the cochlea were not statistically significantly different between the two groups. Dots represent individual values and bars represent the mean ± SD. $p > 0.05$, n = 4.

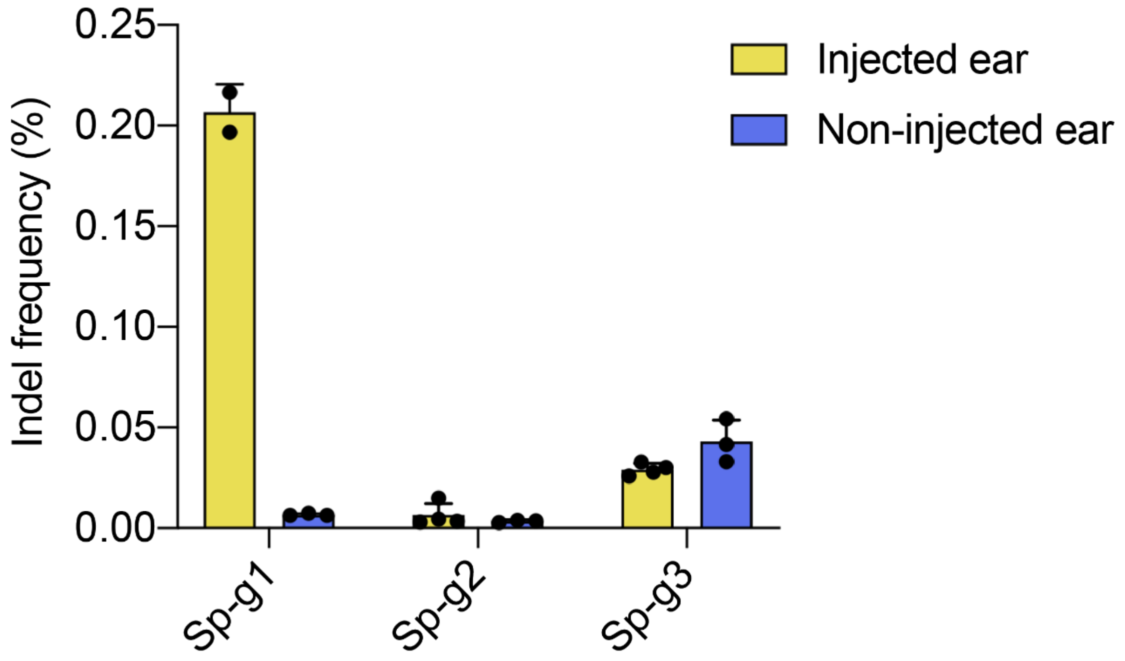


**Fig. S6. *In vivo* genome editing with the Anc80L65–SpCas9 therapeutic system.** The indel frequency of the three gRNAs from injected and non-injected ears *in vivo*. Dots represent individual values and bars represent the mean ± SD, n = 2–4.

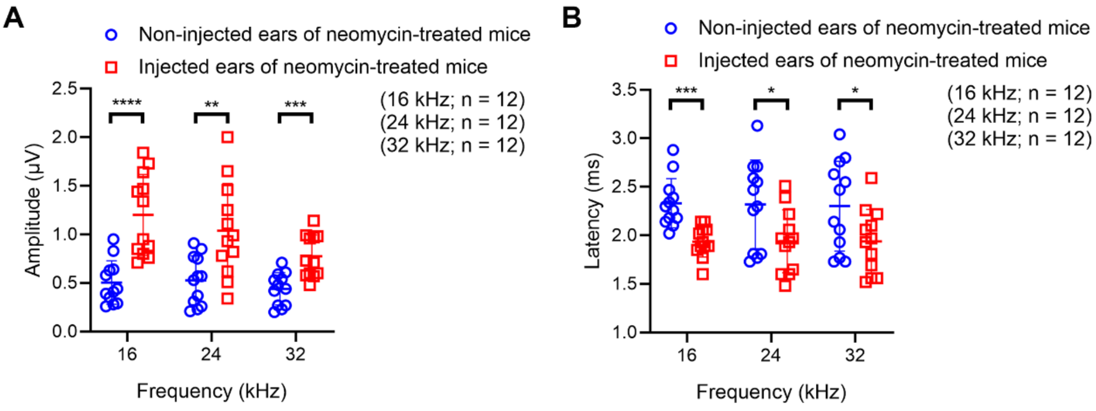


**Fig. S7. Peak amplitudes (A) and latencies (B) of ABR wave 1 evoked by 90 dB SPL at 16–32 kHz in Anc80L65–SpCas9–*Htra2* gRNA-injected ears compared with non-injected ears 4 weeks after the last dose of neomycin.** For statistical analysis, unpaired two-tailed Student's *t*-tests or unpaired *t*-tests with Welch's correction were used as applicable. Individual values are shown; bars represent the mean ± SD. * $p < 0.05$, ** $p < 0.01$, *** $p < 0.001$, **** $p < 0.0001$.

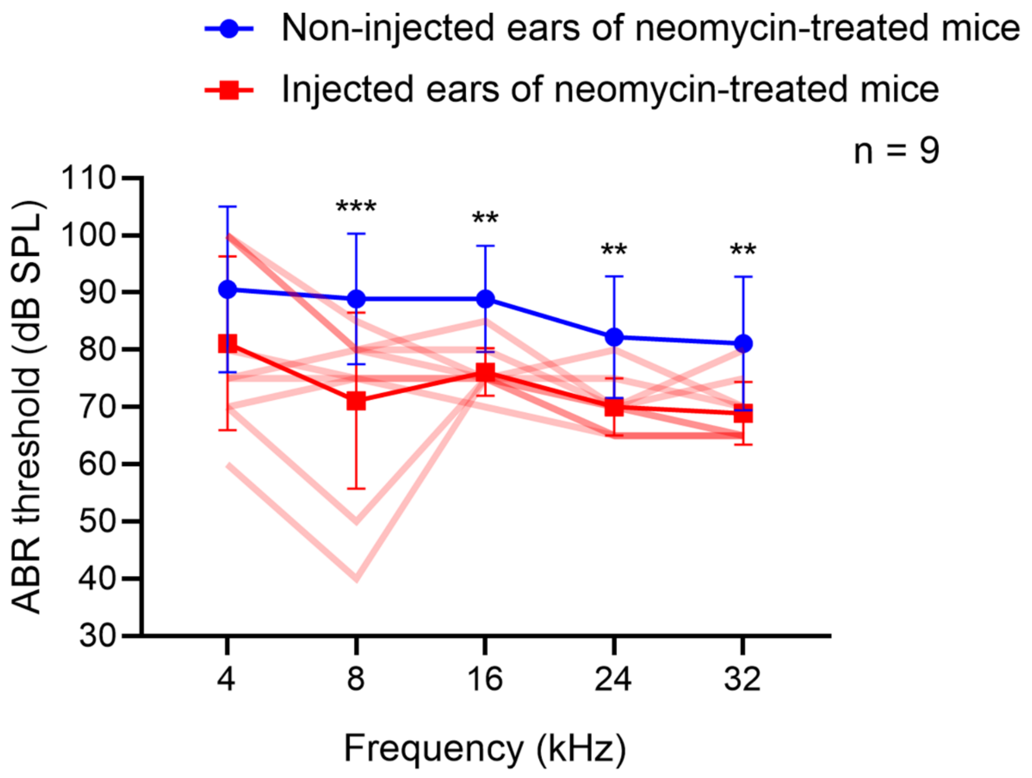


**Fig. S8. Comparison of the ABR thresholds between the non-injected and injected ears 8 weeks after the last dose of neomycin.** For statistical analysis, two-tailed paired *t*-tests or Wilcoxon matched-pairs signed rank tests were used as applicable. Data are presented as the mean ± SD. The lighter red lines represent the ABR thresholds of individual injected ears. ** $p < 0.01$, *** $p < 0.001$.

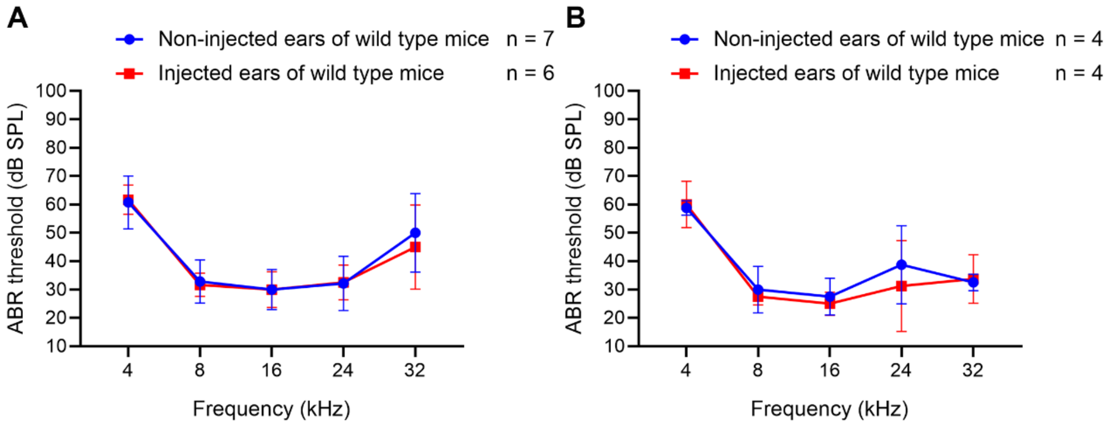


**Fig. S9. The ABR thresholds of non-injected and injected ears of normal mice at different time points after the injection of the Anc80L65–SpCas9 system.** (**A**) Comparison of the ABR thresholds between non-injected and injected ears of normal mice without neomycin exposure at 4 weeks after the injection of the Anc80L65–SpCas9 therapeutic system. (**B**) Comparison of the ABR thresholds between non-injected and injected ears of normal mice without neomycin exposure at 8 weeks after the injection of the Anc80L65–SpCas9 therapeutic system. There was no significant difference in the ABR thresholds between the non-injected and injected ears of normal ICR mice without neomycin damage, which confirms that the injection of Anc80L65–SpCas9–*Htra2* gRNA does not affect the hearing function of wildtype mice. For statistical analysis, unpaired two-tailed Student's *t*-tests or Mann–Whitney *U*-tests were used as applicable. Values and error bars reflect the mean ± SD in **A** and **B**.

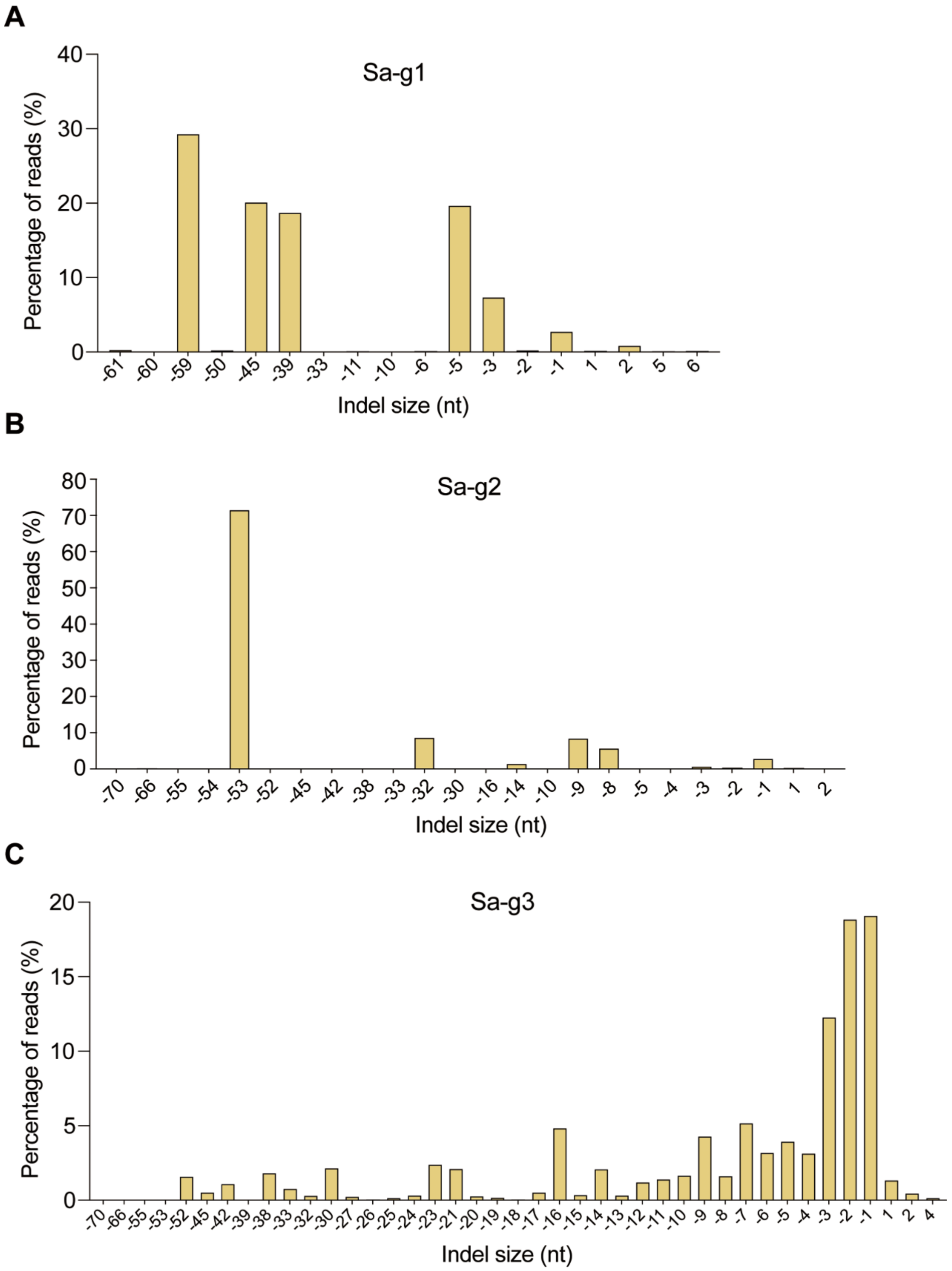


**Fig. S10. Indel profiles with the application of the SaCas9 system *in vitro*.** (**A**–**C**) Indel profiles of Sa-g1 (**A**), Sa-g2 (**B**), and Sa-g3 (**C**) from HEI-OC1 cells transfected with the SaCas9 system. Negative numbers represent deletions, and positive numbers represent insertions.

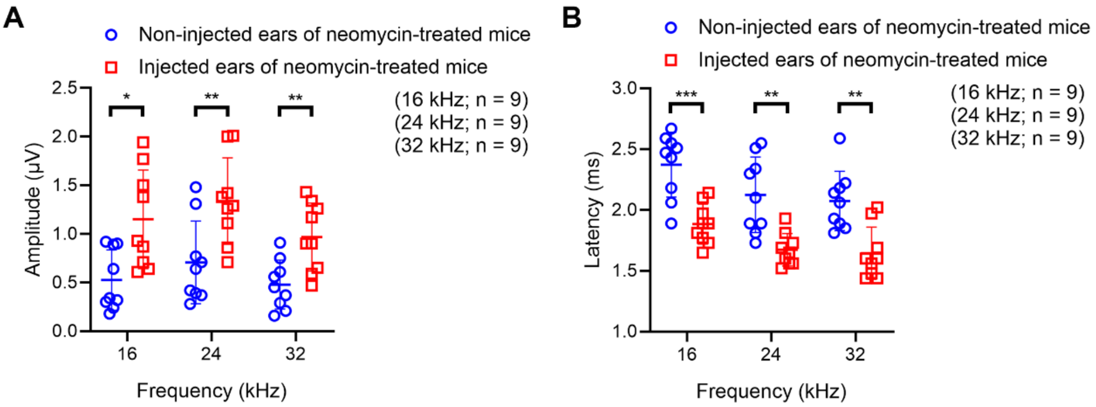


**Fig. S11. Peak amplitudes (A) and latencies (B) of ABR wave 1 evoked by 90 dB SPL at 16–32 kHz in Anc80L65–SaCas9–*Htra2* gRNA-injected ears compared with non-injected ears 2 weeks after the last dose of neomycin.** For statistical analysis, Mann–Whitney *U*-tests, unpaired two-tailed Student's *t*-tests, or unpaired *t*-tests with Welch's correction were used as applicable. Individual values are shown; bars represent the mean ± SD. * $p < 0.05$, ** $p < 0.01$, *** $p < 0.001$.

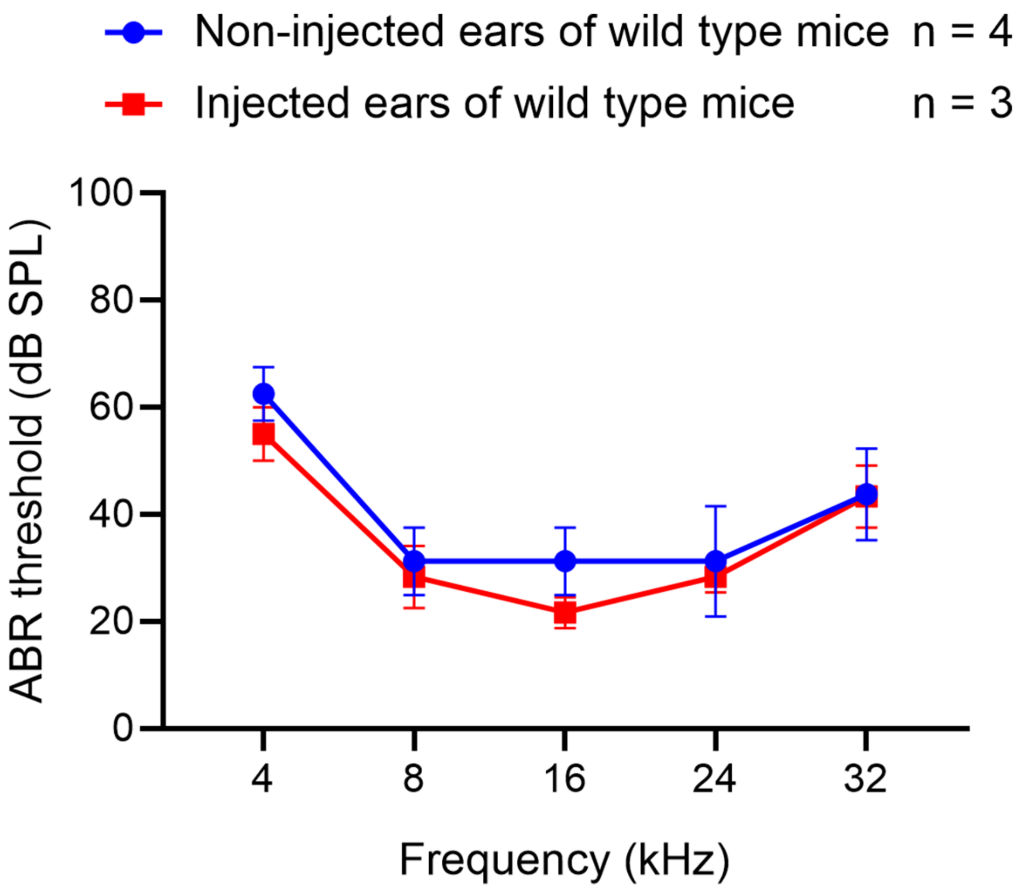


**Fig. S12. Comparison of the ABR thresholds between non-injected and injected ears of normal mice at 4 weeks after injection of Anc80L65–SaCas9–*Htra2* gRNA.** Mann–Whitney *U*-tests were used. Values and error bars reflect the mean ± SD.

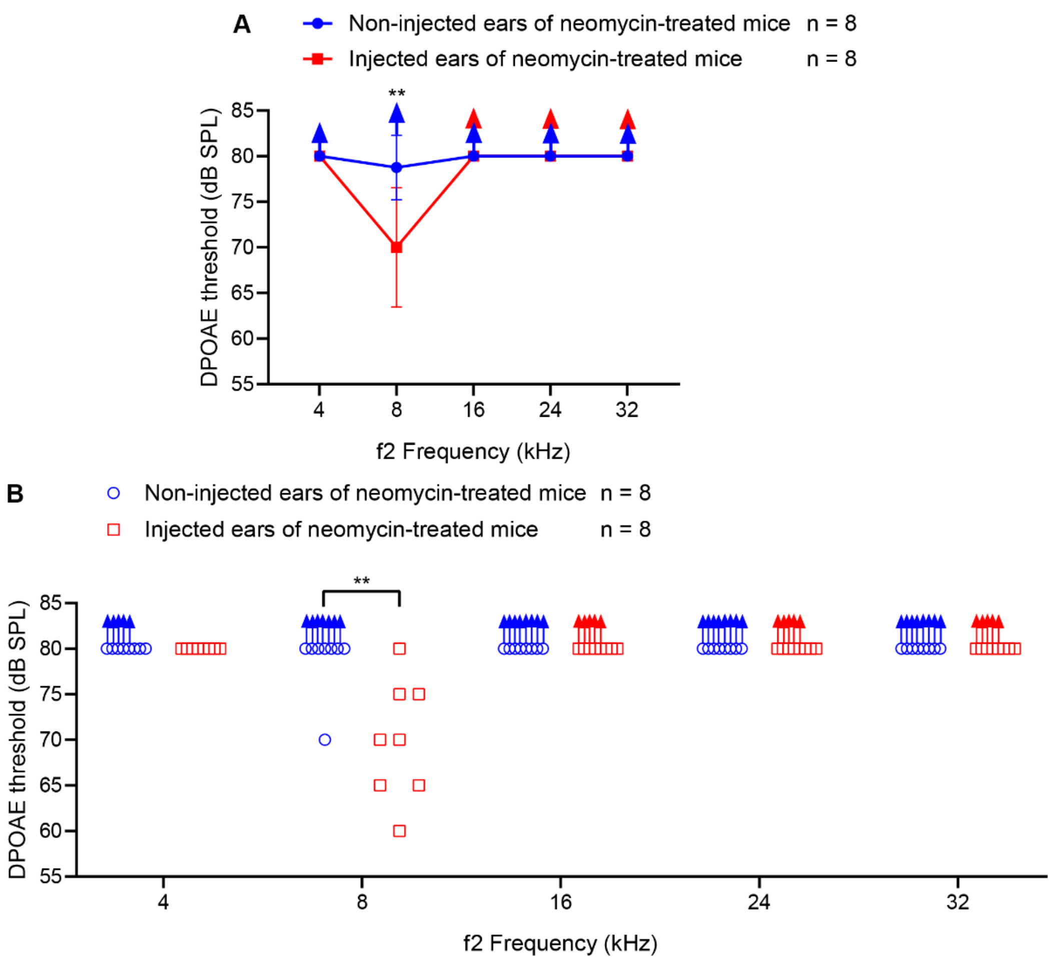


**Fig. S13. Comparison of the DPOAE thresholds between non-injected and injected ears 2 weeks after neomycin exposure with the injection of Anc80L65–SaCas9–*Htra2* gRNA *in vivo*.** (**A**) All the injected ears showed detectable DPOAE thresholds at 4 kHz and 8 kHz. The DPOAE threshold of the injected ears was significantly decreased at 8 kHz compared with the non-injected ears. Data are presented as the mean ± SD. Arrows indicate no detectable DPOAE response at the highest stimulus level tested (80 dB). (**B**) Individual values are shown. Only 37.5% (3/8) and 12.5% (1/8) of the non-injected ears showed detectable DPOAE thresholds following 80 dB SPL stimulation at 4 kHz and 8 kHz respectively, while all the injected ears showed detectable DPOAE thresholds at 4 kHz and 8 kHz. The non-injected ears showed no

detectable DPOAE thresholds following 80 dB SPL stimulation at 16–32 kHz, while 37.5% (3/8) of the injected ears showed detectable DPOAE thresholds at 16 kHz, 24 kHz, and 32 kHz, respectively. Arrows indicate no detectable DPOAE response at the highest stimulus level tested (80 dB). Mann–Whitney *U*-tests were used for statistical analysis. ** $p < 0.01$.
