# Supplementary material for: Prevention of acquired sensorineural hearing loss in mice by in vivo Htra2 gene editing
Source: Genome Biol. 2021 Mar 22;22:86. doi: 10.1186/s13059-021-02311-4 (PMC7983387; doi:10.1186/s13059-021-02311-4)

Full unedited blot for Figure S1B
Western blot result: HtrA2/Omi

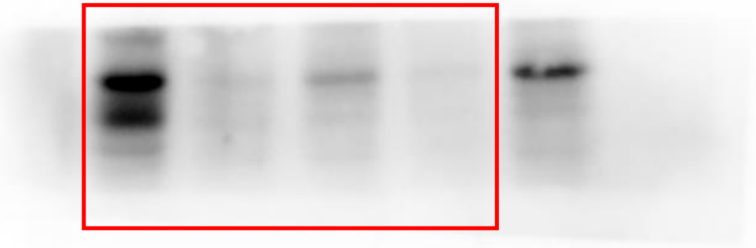

Full unedited blot for Figure S1B
Western blot result: Actin

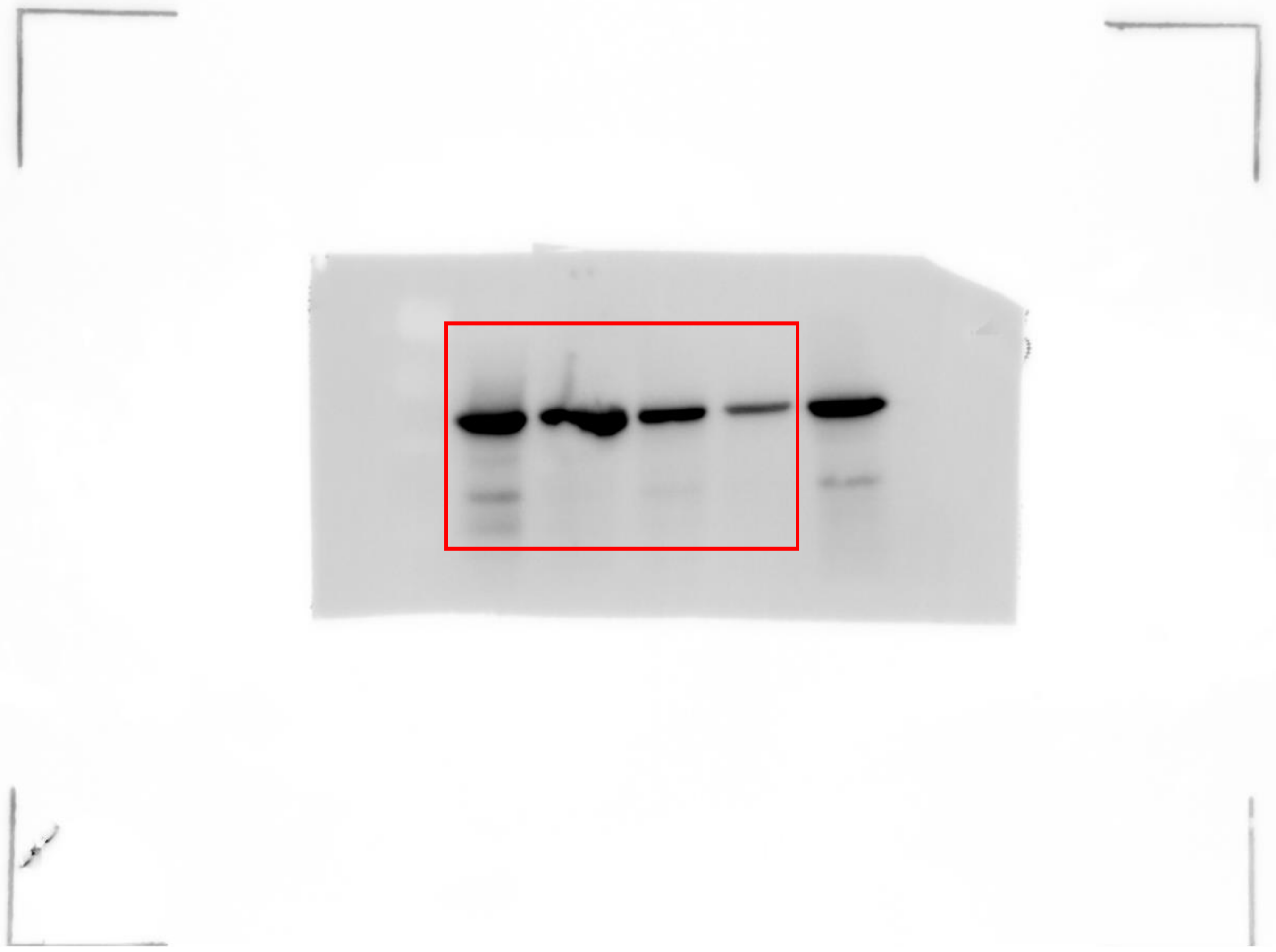

Full unedited gel for Figure S3F

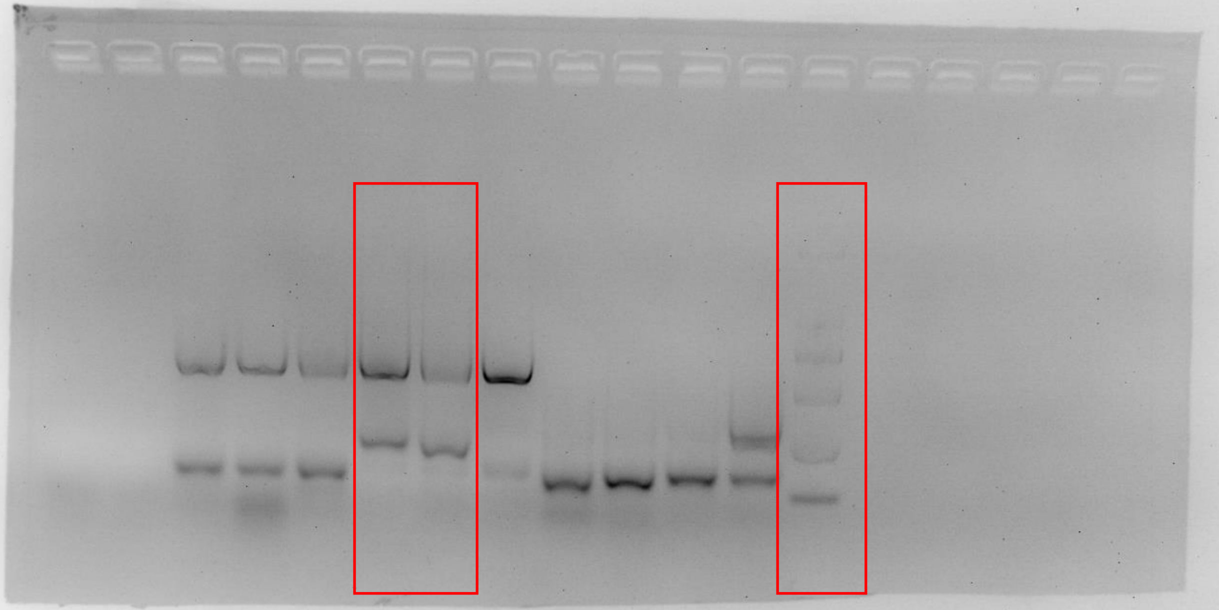

Supplement: Supplementary file 4 — Additional file 4. Source data of blots and gels. [file 13059_2021_2311_MOESM4_ESM.pdf]
