# Supplementary material for: Prevention of acquired sensorineural hearing loss in mice by in vivo Htra2 gene editing
Source: Genome Biol. 2021 Mar 22;22:86. doi: 10.1186/s13059-021-02311-4 (PMC7983387; doi:10.1186/s13059-021-02311-4)
Supplement: Supplementary file 8 — Additional file 8. Review history. [file 13059_2021_2311_MOESM8_ESM.docx]

Review History

**First round of review**

**Reviewer 1**

**Are you able to assess all statistics in the manuscript, including the appropriateness of statistical tests used?** No, I do not feel adequately qualified to assess the statistics.

**Comments to author:**

In this work, Gu and colleagues take an original approach to prevent acquired SNHL by using AAV vectors expressing CRISPR components to knock out a central gene, Htra2. In a mouse model of neomycin-induced deafness, they show fairly efficient and long-term in vivo editing associated with improvements of ABR thresholds in treated mice versus controls.

My only major concern with this work is that the comparison of the Sp versus SaCas9 strategy seems unfair, because SpCas9 was split over two AAVs, whereas the smaller SaCas9 was expressed from a single vector. As correctly pointed out by the authors, the use of a single vector is most likely beneficial, as the splitSpCas9 variant requires equally efficient transduction of the same cell by the two vectors encoding the two halves, which is most likely a rate-limiting step. Thus, I believe the authors' conclusion, that SaCas9 is more efficient in an AAV context, may not be fully supported by the shown data.

Accordingly, if the authors really want to keep the comparison of SpCas9 versus SaCas9 in the paper, the vector design should be adapted to allow for a fairer side-by-side comparison of both Cas9 variants from a single AAV, by e.g. using a smaller promoter and/or omitting the WPRE element (still, the CAG promoter may be too large to drive the full-length SpCas9 cDNA from a single AAV). Other options could be to either remove the SpCas9 part (since the better data are obtained with the SaCas9 version anyways and the paper already comprises a substantial amount of figures and data), or to at least revise the discussion and the conclusion on SaCas9 being better than SpCas9 in an AAV context.

**Reviewer 2**

**Are you able to assess all statistics in the manuscript, including the appropriateness of statistical tests used?** Yes, and I have assessed the statistics in my report.

**Comments to author:**

While several studies over the last few years have used adeno-associated vectors (AAVs) or CRISPR/Cas9 to treat genetic sensorineural hearing loss (SNHL), the current manuscript is one of the first to target acquired SNHL. The authors chose to use such strategies to treat aminoglycoside-induced deafness, a debilitating side effect of certain antimicrobial therapies. They did so by disrupting the Htra2 gene with the help of AAV-mediated intracochlear delivery of CRISPR/SpCas9 and -/SaCas9 systems in neomycin-treated mice. This elegant approach led to reduced apoptosis, increased hair cell survival, and improved hearing function - effects that could be observed for at least several weeks. The manuscript demonstrates the feasibility of treatment of non-inherited hearing loss with the CRISPR/Cas9 technology, which adds to the already existing promising developments and literature in the hearing field. Nevertheless, several points have to be clarified to improve the manuscript and allow the reader to fully grasp the details of every aspect described by the authors.

Major points:

- Lines 545-547, Fig. 2 A, Fig. S1 A and Fig. S3 A: The authors mention in the methods section that different promoters were used for the SaCas9 (CMV) and SpCas9 (CAG) plasmids? They confirm this in Fig. 2 A and Fig. S3 A, where CAG promoters can be seen for the SpCas9 plasmids. Yet, the plasmid draft in Fig. S1 A shows a CMV promoter and SpCas9. The authors subsequently conclude that SaCas9 seemed to be more potent, without mentioning the use of different promoters anywhere else. If indeed CAG and CMV promoters were used, how can one conclude that the differences are not simply due to the choice of promoters?

- Lines 408-410: In addition, the authors write in the discussion: "However, for the therapeutic system, the actual viral dose per cochlea for Anc80L65-SpCas9, Anc80L65-Htra2 gRNA, and Anc80L65-SaCas9-Htra2 gRNA was 1.8 × 10 VG, 0.9 × 10 VG, and 2.7 × 10 VG, respectively." So not even were the promoters different, but also the actual viral doses per cochlea - while it seems logical to conclude the SaCas9 strategy is better than SpCas9 for these experiments (see authors' explanations), several confounders might have played a role in the current manuscript.

- While this does not immediately exclude any use of this approach in humans and does not question the findings of this study, a major issue regarding clinical translatability might be potential consequences of such a therapy for the CNS. To guarantee prevention of potential causes leading to SNHL, the therapy presumably would have to be applied relatively early in life. Several authors have shown patency of the cochlear aqueduct in rodents and at least one study (Landegger et al. Nat Biotechnol 2017, reference 34) has demonstrated AAV expression in the cerebellum after intracochlear application (in mice). While the cochlear aqueduct in adult humans and non-human primates appears to be occluded with fibrous tissue in most cases, the question remains whether there might be a chance of AAVs ending up in the CNS. Specifically, for the strategy outlined in this manuscript, it is relevant that loss of function of Omi/HtrA2 (e.g., triggered by the vectors used by the authors) in the CNS is linked to Parkinson's disease (Strauss et al. Hum Mol Genet. 2005). When describing CRISPR/Cas9-based strategies as "universally applicable as a therapy for this type hearing loss", one would have to mention such possibilities in the discussion/conclusion as well.

- As aminoglycosides damage the vestibular system as well (vestibular ototoxicity), which is another common problem in the clinic, I wonder if the authors pursued any experiments in this direction?

- As the damage is primarily at the level of outer hair cells, were DPOAEs obtained?

Minor points:

ABSTRACT

Good summary of the findings.

BACKGROUND

The introduction is a brief and adequate overview of important factors related to the topic.

Lines 86/87: "Aminoglycosides, such as sisomicin, streptomycin, kanamycin, and neomycin, ..." Considering the importance of the aminoglycoside gentamicin in the medical and hearing field, it would be a good idea to mention it here as one of the most prominent drugs of this class.

Lines 105-107: "Htra2, which encodes a proapoptotic mitochondrial serine protease, induces apoptosis by binding to the apoptosis inhibitory protein XIAP (X-linked inhibitor of apoptosis protein)…" At the moment, it is not clear for a reader unfamiliar with the topic that Omi/HtrA2 is that serin protease, so I would maybe clarify: "The Htra2 gene, which encodes a proapoptotic mitochondrial serine protease (Omi/HtrA2), induces…"

Lines 117-122: "…been applied for the treatment of Duchenne muscular dystrophy, Hutchinson-Gilford progeria syndrome, Leber congenital amaurosis, fragile X syndrome and cancer [22-28]. This advanced technology has also been applied in gene therapy for hereditary hearing loss [29-32], but there have been no reports of the application of this technology in the treatment of acquired non-inherited SNHL." This sounds as if these diseases could routinely be treated with such approaches in humans. Briefly specify in which models what was done. E.g., "…in preclinical studies and first experiments in patients" or add "in mice" when referring to the hearing-specific studies?

RESULTS

Overall, good presentation of data.

- Line 127: "Disruption of the…"

- Lines 129/130: "…SpCas9 with the protospacer-adjacent motif (PAM) sequence of NGG…" Some readers unfamiliar with the technology might be confused, maybe clarify by adding "5'-NGG-3'" or something similar.

- Lines 139/143: Additional file 4 is mentioned before additional file 3?

- Line 310: Typo "SaCa9"

DISCUSSION

While the experimental part is mostly excellent, the authors' discussion needs to include more details.

- Lines 369/370: After discussing AAV therapies for hereditary deafness, the authors state the following without any further explanation: "However, with such a strategy, the functional protein cannot be stably expressed for a long time." In some of these studies, hearing was preserved for multiple months in mice, which would not be the case if the functional protein were to be completely unstable? In addition, human clinical trials with AAVs in other organ systems (e.g., liver or eyes) have demonstrated relatively stable protein expression for decades? Please clarify.

- Lines 370/371: The following sentence also has to be clarified as CRISPR/Cas9 technology is definitely very promising, but has hardly made it into clinical trials (while the above-mentioned AAVs have resulted in several internationally approved therapies): "CRISPR/Cas9 technology can specifically edit target genes and has been used for gene therapy of many diseases."

- Lines 381-383: "We found that the protective effect of the SaCas9 system was better than that of SpCas9 system, and the editing efficiency was higher in the SaCas9 system compared to the SpCas9 system under the same conditions." This sentence potentially has to be revised as well, depending on the authors' findings regarding the promoter/titer (mentioned above).

- Lines 395-397: "A similar corresponding improvement of ABR thresholds would help patients with aminoglycoside-induced hearing loss detect high-decibel sound in the environment and avoid the surgery of cochlear implantation." Not all patients with aminoglycoside-induced hearing loss (vs. deafness) are candidates for cochlear implants, many patients with less extensive auditory deficits can be helped with hearing aids (which is mentioned in the next sentence in the discussion, but one should maybe change the text to "extensive aminogylcoside-induced hearing loss" or similar).

- Lines 404-406: Citing the study by Thorne, Salt et al. from 1999, in which the endolymphatic space of a mouse was approximated with 20µl, appears to be rather outdated. Salt and his group have used more advanced technology in recent years and in a more recent paper from the same laboratory (Hirose et al. J Assoc Res Otolaryngol. 2014), it was stated that: "Total endolymph volume was estimated to be 0.78 µL, made of 0.30 µL in cochlear endolymph and 0.48 µL in the endolymphatic spaces of the vestibular organs."

CONCLUSIONS

Adaptations are needed here as well.

- Lines 432-434: "Given that most acquired non-inherited SNHL is related to apoptosis, CRISPR/Cas9-based strategies might be universally applicable as a therapy for this type hearing loss." While the title of the manuscript and the other sentences of the conclusions correctly talk about prevention of SNHL (which is what was achieved in this study as the CRISPR/Cas9 always was delivered prior to the neomycin trauma), this statement appears to be a bit far-fetched as - if being translatable to the clinic - these strategies would be preventative options for this type of hearing loss, not therapies per se.

METHODS

Elegant study design, methods described sufficiently in most cases.

- Only parts of the "Western blot analysis" paragraph mention catalog numbers (lines 525-531), while the rest of the methods section doesn't?

- Line 553: It is unlikely that just the basilar membrane was cultured, but rather the whole organ of Corti? Were the cochlear explants dissected in a way that the modiolus/neurites/spiral ganglion neuron region was attached to the hair cells or was the organ of Corti cultured separately?

- Lines 559-565: The text says that AAV-treatment groups were incubated in the nutrient solution with AAV vectors for 48 h, then changed to DMEM/F12 mixture without virus, and cultured for another 6 days. Yet in line 566, the authors write the following: "After incubation with Anc80L65-SpCas9 and Anc80L65-gRNA for 8 days…" This is rather confusing for the reader, please clarify.

- Lines 576-580: The authors state that they injected into the scala media - where exactly? Can they be certain about this? Or might have a penetration into one of the other scalae happened in some animals?

- Lines 616-619: When processing the cochlear samples for the in vivo experiments, was transcardial perfusion carried out and/or were round and oval window membranes opened and the inner ear flushed? Or were the whole cochleae with unopened membranes put into 4% PFA for an hour?

- Confocal microscopy: What has not been described in detail so far is whether the laser power/gain/offset were kept identical for any given experiment. For some visualizations/quantifications presented by the authors, this is a crucial point.

REFERENCES

The authors are sometimes not precise enough with their referencing.

- E.g., reference 1 mentions in the abstract that 1.3 billion humans worldwide are affected by SNHL (without citing any source or explaining it anywhere else in the publication). The current manuscript mentions a slightly altered version of the original sentence in the abstract, but just refers the reader to the above-mentioned (incomplete) source. It would be preferable to reference the respective primary literature.

- The text in lines 90/91 talks about sepsis, tuberculosis, respiratory infections in CF, urinary tract infections and endocarditis, yet references 7-9 all only cover bacteremia/sepsis.

FIGURES

The figures support the authors' claims in the text.

- Fig. 1b plots the indel frequency between 70 and 90%. This graph leads readers to overestimate the differences between the three gRNAs when in reality they were "only" up to 15-16%. It is recommended to plot the data from 0 to 90%.

- Figure 1 legend: The abbreviation "nt" has not been defined as nucleotides?

- Figure 2: See comment above, it is unlikely that just the basilar membrane was cultured, so I would change the term "basilar membrane culture". Are both scale bars (in d and e) 20 µm long and apply to all images? The legend should also explain once more that dots represent individual values and bars represent the mean ± SD. For the y-axis label of i, "The" appears to be superfluous, it would appear to be more logical to begin with "Ratio…"?

- Figure 3: Panel b: The graph does not just show mean +- SD, but also individual values (not mentioned in the legend)? Panel c: The legend should mention the Myo7A staining. Panel d: The legend says that "Individual values are shown.", but mean +- SD are shown as well. Panel f: If this indeed shows data at 8 kHz and we have a threshold of 85 dB in the non-injected ear and a threshold of 45 dB in the injected ear, the former is representative while the latter is the single best injected ear (according to the individual results in panel e)? This should be mentioned in the legend and also be reflected in the text in the results section! The legend of panels e and g also doesn't explain that the lighter red lines are the results of individual ears. Panels h and i: Why are amplitude and latency data only presented for 4 and 8 kHz - why not also show the higher frequencies (at least as supplementary data)?

- Figure 4 B shows the injected VG number, which is very helpful and should also be added for the other figures.

- Figure 5: Panel f: See above - the depicted results on the right side seem to be from the single best injected ear (according to the individual results in panel c). This should be mentioned in the legend and also be reflected in the text in the results section! The legend of panels c and g doesn't explain that the lighter red lines are the results of individual ears.

SUPPLEMENTARY DATA

Good additional insights into data analysis.

- Fig. S1 B: While the figure correctly depicts the protein disruption, the substantially different actin bands between loading controls (e.g., Sp-g1 and Sp-g3) are noticeable.

- Fig. S2: Are both scale bars (in A and B) 20 µm long and apply to all images? The legend first talks about the abbreviations "IHCs and OHCs" before using "inner and outer hair cells" - abbreviations should be established when they are mentioned for the first time and then be continuously used.

- Fig. S3: The legend doesn't define the abbreviations for base pairs and nucleotides?

- Fig. S4: Are both scale bars (in A and B) 20 µm long and apply to all images?

- Fig. S5: The legend should explain once more that dots represent individual values and bars represent the mean ± SD.

- Fig. S6: No individual values are depicted, only means and error bars.

- Additional file 2: See above, I would change the term "basilar membrane".

- Fig. S7: The legend doesn't explain that the lighter red lines are the results of individual ears.

- Fig. S8: The legend should clarify that mean +- SD are presented. Is the n=4 for panel B the sum of both groups (n=2 per group) or is the n=4 per group (clarified in panel A, but not panel B)?

**Authors Response**

**Point-by-point responses to the reviewers’ comments:**

Reviewer 1:

In this work, Gu and colleagues take an original approach to prevent acquired SNHL by using AAV vectors expressing CRISPR components to knock out a central gene, Htra2. In a mouse model of neomycin-induced deafness, they show fairly efficient and long-term in vivo editing associated with improvements of ABR thresholds in treated mice versus controls.

My only major concern with this work is that the comparison of the Sp versus SaCas9 strategy seems unfair, because SpCas9 was split over two AAVs, whereas the smaller SaCas9 was expressed from a single vector. As correctly pointed out by the authors, the use of a single vector is most likely beneficial, as the splitSpCas9 variant requires equally efficient transduction of the same cell by the two vectors encoding the two halves, which is most likely a rate-limiting step. Thus, I believe the authors' conclusion, that SaCas9 is more efficient in an AAV context, may not be fully supported by the shown data.

Accordingly, if the authors really want to keep the comparison of SpCas9 versus SaCas9 in the paper, the vector design should be adapted to allow for a fairer side-by-side comparison of both Cas9 variants from a single AAV, by e.g. using a smaller promoter and/or omitting the WPRE element (still, the CAG promoter may be too large to drive the full-length SpCas9 cDNA from a single AAV). Other options could be to either remove the SpCas9 part (since the better data are obtained with the SaCas9 version anyways and the paper already comprises a substantial amount of figures and data), or to at least revise the discussion and the conclusion on SaCas9 being better than SpCas9 in an AAV context.

*Author’s response to the comments: Thanks for your comments. We agree with the comment that the comparison of the SpCas9 versus SaCas9 strategy seems unfair and have made corresponding revision in the discussion section. Since the splitSpCas9 variant requires equally efficient transduction of the same cell by the two vectors encoding the two halves, the use of a single vector encoding the SaCas9 system is most likely beneficial. However, with our present data, we cannot draw a conclusion that SaCas9 is better than SpCas9 in an AAV context, because the vector design in our work does not allow for a fair side-by-side comparison of both Cas9 variants from a single AAV vector. The conclusion, that SaCas9 is more efficient than SpCas9 in an AAV context, has been removed from the text. We have re-written this part according to your suggestion as follows.*

*In the current study, we used two CRISPR/Cas9 systems, SpCas9 and SaCas9, to knock out the Htra2 gene, and both systems reached the goal of hearing protection to some extent. It is exceedingly difficult to package SpCas9 into a single AAV2/Anc80L65 vector due to its limited packing capacity, but we successfully constructed Anc80L65–SpCas9 system with the split-SpCas9 scheme in the field of gene therapy for hearing loss. However, the delivery of the SpCas9 system using three AAV vectors might have affected the transduction efficiency of the whole therapeutic system in hair cells, which may be one of the factors leading to lower editing efficiency compared with the SaCas9 system. Moreover, the different promoters and viral doses used for the SpCas9 and SaCas9 system might be other key confounders leading to different therapeutic effects between the two systems.*

Reviewer 2:

While several studies over the last few years have used adeno-associated vectors (AAVs) or CRISPR/Cas9 to treat genetic sensorineural hearing loss (SNHL), the current manuscript is one of the first to target acquired SNHL. The authors chose to use such strategies to treat aminoglycoside-induced deafness, a debilitating side effect of certain antimicrobial therapies. They did so by disrupting the Htra2 gene with the help of AAV-mediated intracochlear delivery of CRISPR/SpCas9 and -/SaCas9 systems in neomycin-treated mice. This elegant approach led to reduced apoptosis, increased hair cell survival, and improved hearing function - effects that could be observed for at least several weeks. The manuscript demonstrates the feasibility of treatment of non-inherited hearing loss with the CRISPR/Cas9 technology, which adds to the already existing promising developments and literature in the hearing field. Nevertheless, several points have to be clarified to improve the manuscript and allow the reader to fully grasp the details of every aspect described by the authors.

Major points:

- Lines 545-547, Fig. 2 A, Fig. S1 A and Fig. S3 A: The authors mention in the methods section that different promoters were used for the SaCas9 (CMV) and SpCas9 (CAG) plasmids? They confirm this in Fig. 2 A and Fig. S3 A, where CAG promoters can be seen for the SpCas9 plasmids. Yet, the plasmid draft in Fig. S1 A shows a CMV promoter and SpCas9. The authors subsequently conclude that SaCas9 seemed to be more potent, without mentioning the use of different promoters anywhere else. If indeed CAG and CMV promoters were used, how can one conclude that the differences are not simply due to the choice of promoters?

*Author’s response to the comments: Thanks for your comments. We indeed used different promoters for the SaCas9 (CMV) and SpCas9 (CAG) plasmids. Only in the initial study of testing the indel frequencies of the three gRNAs (Sp-g1, Sp-g2, and Spg3), we used a CMV promoter for the SpCas9 system (Fig. S1 A). Then we examined the in vitro genome editing in HEI-OC1 cells with the SpCas9 (CAG) system in which the three gRNAs were constructed in one plasmid to knock out the Htra2 gene more efficiently (Fig. S3 A). Considering the different promoters were used in the SpCas9 and SaCas9 system, we cannot conclude that SaCas9 seemed to be more potent. The relevant statements have been removed from the text or corrected in the manuscript. We have deleted the following sentences:*

*“We found that the protective effect of the SaCas9 system was better than that of SpCas9 system, and the editing efficiency was higher in the SaCas9 system compared to the SpCas9 system under the same conditions.”*

*“Our data suggest that the CRISPR/SaCas9 system may be a better choice when AAVs are used as the delivery vector for genome editing of a single target.”*

*And we have added the following sentence:*

*“Moreover, the different promoters and viral doses used for the SpCas9 and SaCas9 system might be other key confounders leading to different therapeutic effects between the two systems.”*

- Lines 408-410: In addition, the authors write in the discussion: "However, for the therapeutic system, the actual viral dose per cochlea for Anc80L65-SpCas9, Anc80L65-Htra2 gRNA, and Anc80L65-SaCas9-Htra2 gRNA was 1.8 × 10 VG, 0.9 × 10 VG, and 2.7 × 10 VG, respectively." So not even were the promoters different, but also the actual viral doses per cochlea - while it seems logical to conclude the SaCas9 strategy is better than SpCas9 for these experiments (see authors' explanations), several confounders might have played a role in the current manuscript.

*Author’s response to the comments: As you pointed out in the comment, the different viral doses for SpCas9 and SaCas9 system might be another important confounder leading to different therapeutic effects between the two systems. So, we have removed the conclusion, that SaCas9 is more efficient than SpCas9 in an AAV context, from the manuscript.*

- While this does not immediately exclude any use of this approach in humans and does not question the findings of this study, a major issue regarding clinical translatability might be potential consequences of such a therapy for the CNS. To guarantee prevention of potential causes leading to SNHL, the therapy presumably would have to be applied relatively early in life. Several authors have shown patency of the cochlear aqueduct in rodents and at least one study (Landegger et al. Nat Biotechnol 2017, reference 34) has demonstrated AAV expression in the cerebellum after intracochlear application (in mice). While the cochlear aqueduct in adult humans and non-human primates appears to be occluded with fibrous tissue in most cases, the question remains whether there might be a chance of AAVs ending up in the CNS. Specifically, for the strategy outlined in this manuscript, it is relevant that loss of function of Omi/HtrA2 (e.g., triggered by the vectors used by the authors) in the CNS is linked to Parkinson's disease (Strauss et al. Hum Mol Genet. 2005). When describing CRISPR/Cas9-based strategies as "universally applicable as a therapy for this type hearing loss", one would have to mention such possibilities in the discussion/conclusion as well.

*Author’s response to the comments: This suggestion is especially important to the clinical translatability of our AAV–CRISPR/Cas9–Htra2 gRNA strategy. Omi/HtrA2 is a proapoptotic mitochondrial serine protease that is released into the cytoplasm following apoptotic insult (Kilbride, S. M. & Prehn, J. H. Oncogene. 2013). Wang et al. observed that increased expression of Omi/HtrA2 in aging rats augmented myocardial ischemia/reperfusion injury by stimulating myocardial apoptosis (Wang et al. Age. 2013), which suggests that strategies to inhibit Omi/HtrA2 may protect against heart injury. However, a neurodegenerative phenotype with parkinsonian features has been defined in Omi/HtrA2 knockout mice (Martins et al. Mol Cell Biol. 2004), and Strauss et al. performed a mutation screening of the Omi/HtrA2 gene in German Parkinson’s disease (PD) patients and identified a heterozygous G399S mutation in four (4/518) patients (Strauss et al. Hum Mol Genet. 2005), which indicates that loss of function of Omi/HtrA2 in the central nervous system (CNS) may be linked to PD. The pathological hallmark of PD is depigmentation of the substantia nigra and locus coeruleus with neuronal loss in the pars compacta of the substantia nigra (Hayes et al. Am J Med. 2019). In mice, the cochlear aqueduct is patent, providing a fluid path from the cochlear perilymph into the cerebrospinal fluid. Landegger et al. has demonstrated that Anc80L65-eGFP injected via round window membrane transduced Purkinje neurons in the cerebellum (Landegger et al. Nat Biotechnol. 2017) which is not the affected brain area in PD. In our work, the AAV–CRISPR/Cas9 system was injected into the scala media of the cochlea. As safety assessment is a particularly important aspect of gene therapy, it is necessary to examine whether the therapeutic system enters into the CNS and which encephalic regions and types of neurons are transduced with such injection route in future study. Together, to take a further step towards clinical translation of this CRISPR/Cas9-based strategy, it requires further investigation to evaluate the potential influence of this treatment on the CNS. The above statement has been added in the discussion section.*

- As aminoglycosides damage the vestibular system as well (vestibular ototoxicity), which is another common problem in the clinic, I wonder if the authors pursued any experiments in this direction?

*Author’s response to the comments: In this study, no mice developed any behavioural signs of vestibular damage after neomycin exposure in vivo. We assume that the neomycin-damage pattern used in our study was insufficient to cause vestibular dysfunction in mice. At P11, mice received a daily subcutaneous injection of neomycin (200 mg/kg) continuously for 7 days. Within the observation period, neomycin-exposed mice showed no obvious symptoms of postural asymmetries, including head deviation, trunk curvature, forelimb extension, circling compulsory movements, and head nystagmus. Moreover, Tsuji et al. (Ann Otol Rhinol Laryngol. 2000) performed a quantitative assessment of vestibular hair cells and Scarpa's ganglion cells in temporal bones from patients who had aminoglycoside ototoxicity (streptomycin, kanamycin, and neomycin). For neomycin, the data in their study suggested that there was little hair cell ototoxic effect in the vestibular sense organs. Aminoglycosides damage the vestibular system as well, which is another common problem in the clinic. It was reported that the severity of vestibular damage was in the order of streptomycin, gentamicin, amikacin, and netilmicin in Guinea pigs (Selimoglu et al. Yonsei Med J. 2003). Therefore, it is meaningful to investigate whether the AAV–CRISPR/Cas9 strategy protects the vestibular system from aminoglycoside-induced ototoxicity in future study.*

*The above statement has been added in the discussion section.*

- As the damage is primarily at the level of outer hair cells, were DPOAEs obtained?

*Author’s response to the comments: We have supplemented the DPOAE experiments and added the results and methods in the relevant sections.*

*The content of the DPOAE part is as follows:*

*We also measured distortion product otoacoustic emissions (DPOAEs) which represent the amplification provided by OHCs. Two weeks after the last dose of neomycin, only 37.5% (3/8) and 12.5% (1/8) of the non-injected ears showed detectable DPOAE thresholds following 80 dB SPL stimulation at 4 kHz and 8 kHz respectively, while all the injected ears showed detectable DPOAE thresholds at 4 kHz and 8 kHz (Additional file 1: Fig. S13). The DPOAE threshold of the Anc80L65–SaCas9–Htra2 gRNA-injected ears was significantly decreased at 8 kHz compared with the non-injected ears (Additional file 6). The non-injected ears showed no detectable DPOAE thresholds following 80 dB SPL stimulation at middle and high frequency ranges (16–32 kHz), consistent with the severe OHC damage in the corresponding regions, while 37.5% (3/8) of the injected ears showed detectable DPOAE thresholds at 16 kHz, 24 kHz, and 32 kHz, respectively (Additional file 1: Fig. S13). Although the ABR thresholds of the injected ears at 16–32 kHz were decreased significantly compared to the non-injected ears (16 kHz, 24 kHz, and 32 kHz: injected, 76.7 ± 5.0 dB, 75.0 ± 9.0 dB, and 71.1 ± 7.0 dB, respectively; non-injected, 86.7 ± 9.4 dB, 87.2 ± 7.1 dB, and 86.1 ± 8.9 dB, respectively, n = 9, p < 0.05), the injected ears did not present conspicuous DPOAE recovery at the corresponding frequencies. We consider that the improvement of the ABR thresholds at 16–32 kHz in the injected ears may be mainly due to the protection of IHCs in the corresponding regions. Together, these results indicate that injection of Anc80L65–SaCas9–Htra2 gRNA into the inner ears of neonatal mice partially rescues the hearing function after neomycin exposure in vivo.*

Minor points:

ABSTRACT

Good summary of the findings.

*Author’s response to the comments: Thank you for the affirmation*

BACKGROUND

The introduction is a brief and adequate overview of important factors related to the topic.

Lines 86/87: "Aminoglycosides, such as sisomicin, streptomycin, kanamycin, and neomycin, ..." Considering the importance of the aminoglycoside gentamicin in the medical and hearing field, it would be a good idea to mention it here as one of the most prominent drugs of this class.

*Author’s response to the comments: The “gentamicin” has been added in the text as*

*you suggested.*

Lines 105-107: "Htra2, which encodes a proapoptotic mitochondrial serine protease, induces apoptosis by binding to the apoptosis inhibitory protein XIAP (X-linked inhibitor of apoptosis protein)…" At the moment, it is not clear for a reader unfamiliar with the topic that Omi/HtrA2 is that serin protease, so I would maybe clarify: "The Htra2 gene, which encodes a proapoptotic mitochondrial serine protease (Omi/HtrA2), induces…"

*Author’s response to the comments: The sentence has been revised as you indicated.*

Lines 117-122: "…been applied for the treatment of Duchenne muscular dystrophy, Hutchinson-Gilford progeria syndrome, Leber congenital amaurosis, fragile X syndrome and cancer [22-28]. This advanced technology has also been applied in gene therapy for hereditary hearing loss [29-32], but there have been no reports of the application of this technology in the treatment of acquired non-inherited SNHL." This sounds as if these diseases could routinely be treated with such approaches in humans. Briefly specify in which models what was done. E.g., "…in preclinical studies and first experiments in patients" or add "in mice" when referring to the hearing-specific studies?

Author’s response to the comments: The statement has been revised as you indicated.

RESULTS

Overall, good presentation of data.

- Line 127: "Disruption of the…"

*Author’s response to the comments: The subtitle has been revised.*

- Lines 129/130: "…SpCas9 with the protospacer-adjacent motif (PAM) sequence of NGG…" Some readers unfamiliar with the technology might be confused, maybe clarify by adding "5'-NGG-3'" or something similar.

*Author’s response to the comments: The sentence has been revised.*

- Lines 139/143: Additional file 4 is mentioned before additional file 3?

*Author’s response to the comments: The order of “additional file 3” and “additional file*

*4” has been corrected in the text.*

- Line 310: Typo "SaCa9"

*Author’s response to the comments: It has been corrected in the text.*

DISCUSSION

While the experimental part is mostly excellent, the authors' discussion needs to include more details.

- Lines 369/370: After discussing AAV therapies for hereditary deafness, the authors state the following without any further explanation: "However, with such a strategy, the functional protein cannot be stably expressed for a long time." In some of these studies, hearing was preserved for multiple months in mice, which would not be the case if the functional protein were to be completely unstable? In addition, human clinical trials with AAVs in other organ systems (e.g., liver or eyes) have demonstrated relatively stable protein expression for decades? Please clarify.

*Author’s response to the comments: As you indicated in this comment, it is inappropriate to state "However, with such a strategy, the functional protein cannot be stably expressed for a long time". We have removed this sentence from the text and re-written this part as follows:*

*Transfer of the therapeutic wild-type genes offers a potential treatment strategy for recessive genetic diseases, but this approach may require repeated administration to obtain a lifetime therapeutic effect, which increases the risk of inducing side effects, including clinically relevant immunogenicity (Halbert et al. J Virol. 2000). CRISPR/Cas9 technology can specifically edit target genes and offers promising alternatives for diseases such as Duchenne muscular dystrophy (Amoasii et al. Sci Transl Med. 2017), metabolopathies (Yang et al. Nat Biotechnol. 2016) and deafness (Gao et al. Nature. 2018), providing a potential one-time treatment strategy for genetic diseases.*

- Lines 370/371: The following sentence also has to be clarified as CRISPR/Cas9 technology is definitely very promising, but has hardly made it into clinical trials (while the above-mentioned AAVs have resulted in several internationally approved therapies): "CRISPR/Cas9 technology can specifically edit target genes and has been used for gene therapy of many diseases."

*Author’s response to the comments: The statement has been revised as follows:*

*CRISPR/Cas9 technology can specifically edit target genes and offers promising alternatives for diseases such as Duchenne muscular dystrophy (Amoasii et al. Sci Transl Med. 2017), metabolopathies (Yang et al. Nat Biotechnol. 2016) and deafness (Gao et al. Nature. 2018), providing a potential one-time treatment strategy for genetic diseases.*

- Lines 381-383: "We found that the protective effect of the SaCas9 system was better than that of SpCas9 system, and the editing efficiency was higher in the SaCas9 system compared to the SpCas9 system under the same conditions." This sentence potentially has to be revised as well, depending on the authors' findings regarding the promoter/titer (mentioned above).

*Author’s response to the comments: This sentence has been removed from the text.*

- Lines 395-397: "A similar corresponding improvement of ABR thresholds would help patients with aminoglycoside-induced hearing loss detect high-decibel sound in the environment and avoid the surgery of cochlear implantation." Not all patients with aminoglycoside-induced hearing loss (vs. deafness) are candidates for cochlear implants, many patients with less extensive auditory deficits can be helped with hearing aids (which is mentioned in the next sentence in the discussion, but one should maybe change the text to "extensive aminogylcoside-induced hearing loss" or similar).

*Author’s response to the comments: The sentence has been revised as you suggested.*

- Lines 404-406: Citing the study by Thorne, Salt et al. from 1999, in which the endolymphatic space of a mouse was approximated with 20µl, appears to be rather outdated. Salt and his group have used more advanced technology in recent years and in a more recent paper from the same laboratory (Hirose et al. J Assoc Res Otolaryngol. 2014), it was stated that: "Total endolymph volume was estimated to be 0.78 µL, made of 0.30 µL in cochlear endolymph and 0.48 µL in the endolymphatic spaces of the vestibular organs."

*Author’s response to the comments: The endolymph volume has been revised to 0.78 µl, and the corresponding reference has been updated to the more recent paper.*

CONCLUSIONS

Adaptations are needed here as well.

- Lines 432-434: "Given that most acquired non-inherited SNHL is related to apoptosis, CRISPR/Cas9-based strategies might be universally applicable as a therapy for this type hearing loss." While the title of the manuscript and the other sentences of the conclusions correctly talk about prevention of SNHL (which is what was achieved in this study as the CRISPR/Cas9 always was delivered prior to the neomycin trauma), this statement appears to be a bit far-fetched as - if being translatable to the clinic - these strategies would be preventative options for this type of hearing loss, not therapies per se.

*Author’s response to the comments: The statement has been revised according to your suggestion.*

METHODS

Elegant study design, methods described sufficiently in most cases.

- Only parts of the "Western blot analysis" paragraph mention catalog numbers (lines 525-531), while the rest of the methods section doesn't?

*Author’s response to the comments: The catalog number for each agent has been added in the methods section.*

- Line 553: It is unlikely that just the basilar membrane was cultured, but rather the whole organ of Corti? Were the cochlear explants dissected in a way that the modiolus/neurites/spiral ganglion neuron region was attached to the hair cells or was the organ of Corti cultured separately?

*Author’s response to the comments: Actually, the whole organ of Corti was cultured. The words of “basilar membrane” have been changed to “organ of Corti”. The organ of Corti was cultured separately without the modiolus/neurites/spiral ganglion neuron region attached to the hair cells.*

- Lines 559-565: The text says that AAV-treatment groups were incubated in the nutrient solution with AAV vectors for 48 h, then changed to DMEM/F12 mixture without virus, and cultured for another 6 days. Yet in line 566, the authors write the following: "After incubation with Anc80L65-SpCas9 and Anc80L65-gRNA for 8 days…" This is rather confusing for the reader, please clarify.

*Author’s response to the comments: "After incubation with Anc80L65-SpCas9 and Anc80L65-gRNA for 8 days…" has been revised to “After total 8 days of culture (2 days with virus and 6 days without virus)”.*

- Lines 576-580: The authors state that they injected into the scala media - where exactly? Can they be certain about this? Or might have a penetration into one of the other scalae happened in some animals?

*Author’s response to the comments: About the injection method, we referred to the study by Wang et al. (J Gene Med. 2013). In Figure 1 A in their study, an illustration of the surgical approach and landmarks used during surgery performed at P2 was shown. The basal turn of the cochlea (the site of injection) can be seen above the stapedius artery. We consider that the possibility of a penetration into one of the other scalae in some animals might be small.*

*The reference has been added in the manuscript.*

- Lines 616-619: When processing the cochlear samples for the in vivo experiments, was transcardial perfusion carried out and/or were round and oval window membranes opened and the inner ear flushed? Or were the whole cochleae with unopened membranes put into 4% PFA for an hour?

*Author’s response to the comments: When processing the cochlear samples for the in vivo experiments, we did not carry out transcardial perfusion. The round and oval window membranes were opened, and the inner ears were flushed with 4% PFA. This sentence has been added in the manuscript.*

- Confocal microscopy: What has not been described in detail so far is whether the laser power/gain/offset were kept identical for any given experiment. For some visualizations/quantifications presented by the authors, this is a crucial point.

*Author’s response to the comments: The scanning parameters of confocal microscope, including laser power, smart gain, and smart offset, were kept identical across different samples which were stained with the same antibodies and under the same staining conditions. This sentence has been added in the manuscript.*

REFERENCES

The authors are sometimes not precise enough with their referencing.

- E.g., reference 1 mentions in the abstract that 1.3 billion humans worldwide are affected by SNHL (without citing any source or explaining it anywhere else in the publication). The current manuscript mentions a slightly altered version of the original sentence in the abstract, but just refers the reader to the above-mentioned (incomplete) source. It would be preferable to reference the respective primary literature.

*Author’s response to the comments: The respective primary literature has been added in the references, and the old reference 1 has been changed to the new one.*

- The text in lines 90/91 talks about sepsis, tuberculosis, respiratory infections in CF, urinary tract infections and endocarditis, yet references 7-9 all only cover bacteremia/sepsis.

*Author’s response to the comments: The references concerning tuberculosis, respiratory infections in cystic fibrosis, urinary tract infections and endocarditis have been supplemented in the manuscript.*

FIGURES

The figures support the authors' claims in the text.

- Fig. 1b plots the indel frequency between 70 and 90%. This graph leads readers to overestimate the differences between the three gRNAs when in reality they were "only" up to 15-16%. It is recommended to plot the data from 0 to 90%.

*Author’s response to the comments: Fig. 1b has been revised as you recommended.*

- Figure 1 legend: The abbreviation "nt" has not been defined as nucleotides?

*Author’s response to the comments: The abbreviation "nt" has been defined as*

*nucleotides now in the legend.*

- Figure 2: See comment above, it is unlikely that just the basilar membrane was cultured, so I would change the term "basilar membrane culture". Are both scale bars (in d and e) 20 µm long and apply to all images? The legend should also explain once more that dots represent individual values and bars represent the mean ± SD. For the y-axis label of i, "The" appears to be superfluous, it would appear to be more logical to begin with "Ratio…"?

*Author’s response to the comments: The term "basilar membrane culture" has been*

*changed to “organ of Corti culture”. Both scale bars (in d and e) represent 20 µm long*

*and apply to all images. This sentence has been added in the legend. We also add the*

*explanations as you suggested. “The ratio” has been changed to “Ratio”.*

- Figure 3: Panel b: The graph does not just show mean +- SD, but also individual values (not mentioned in the legend)? Panel c: The legend should mention the Myo7A staining. Panel d: The legend says that "Individual values are shown.", but mean +- SD are shown as well. Panel f: If this indeed shows data at 8 kHz and we have a threshold of 85 dB in the non-injected ear and a threshold of 45 dB in the injected ear, the former is representative while the latter is the single best injected ear (according to the individual results in panel e)? This should be mentioned in the legend and also be reflected in the text in the results section! The legend of panels e and g also doesn't explain that the lighter red lines are the results of individual ears. Panels h and i: Why are amplitude and latency data only presented for 4 and 8 kHz - why not also show the higher frequencies (at least as supplementary data)?

*Author’s response to the comments: Panel b: “The mean ± SD indel frequency of the three gRNAs” has been revised as “The individual (dot) and mean ± SD (bar) indel frequency of the three gRNAs”. Panel c: Representative confocal images showing immunofluorescence staining with Myo7a (red) 2 weeks after the last dose of neomycin from an injected cochlea and the contralateral (non-injected) cochlea. Panel d: Individual values are shown; bars represent the mean ± SD. Panel f: This has been mentioned in the legend and reflected in the text in the results section as you suggested. Panels e and g: The sentence has been added in the legend: “The lighter red lines in e and g represent the ABR thresholds of individual injected ears”. Panels h and i: The amplitude and latency data for the higher frequencies have been supplemented in Additional file 1: Figure S7.*

- Figure 4 B shows the injected VG number, which is very helpful and should also be added for the other figures.

*Author’s response to the comments: The incubated VG number for the SpCas9 system in vitro has been added in Figure 2. The injected VG number for the SpCas9 system in vivo has been added in Figure 3.*

- Figure 5: Panel f: See above - the depicted results on the right side seem to be from the single best injected ear (according to the individual results in panel c). This should be mentioned in the legend and also be reflected in the text in the results section! The legend of panels c and g doesn't explain that the lighter red lines are the results of individual ears.

*Author’s response to the comments: Panel f: This has been mentioned in the legend and reflected in the text in the results section as you suggested. Panels c and g: The sentence has been added in the legend: “The lighter red lines in c and g represent the ABR thresholds of individual injected ears”. The amplitude and latency data for the higher frequencies have been supplemented in Additional file 1: Figure S11.*

SUPPLEMENTARY DATA

Good additional insights into data analysis.

- Fig. S1 B: While the figure correctly depicts the protein disruption, the substantially different actin bands between loading controls (e.g., Sp-g1 and Sp-g3) are noticeable.

*Author’s response to the comments: The substantially different actin bands between*

*loading controls, especially between Sp-g1 and Sp-g3, were due to relatively few cells*

*in Sp-g3 group. So, we analyzed the gray values of bands from Omi/HtrA2 (target*

*protein) and actin (reference protein) and used protein relative expression to present*

*the protein disruption.*

- Fig. S2: Are both scale bars (in A and B) 20 µm long and apply to all images? The legend first talks about the abbreviations "IHCs and OHCs" before using "inner and outer hair cells" - abbreviations should be established when they are mentioned for the first time and then be continuously used.

*Author’s response to the comments: Both scale bars (in A and B) represent 20 µm long and apply to all images. This sentence has been added in the legend. The problem of abbreviations has been solved according to your instructions.*

- Fig. S3: The legend doesn't define the abbreviations for base pairs and nucleotides?

*Author’s response to the comments: The abbreviations for base pairs and nucleotides are defined now.*

- Fig. S4: Are both scale bars (in A and B) 20 µm long and apply to all images?

*Author’s response to the comments: Both scale bars (in A and B) represent 20 µm long and apply to all images. This sentence has been added in the legend.*

- Fig. S5: The legend should explain once more that dots represent individual values and bars represent the mean ± SD.

*Author’s response to the comments: The explanation has been added in the legend.*

- Fig. S6: No individual values are depicted, only means and error bars.

*Author’s response to the comments: The individual values have been added in the*

*figure.*

- Additional file 2: See above, I would change the term "basilar membrane".

*Author’s response to the comments: The term "basilar membrane" has been changed to “organ of Corti”.*

- Fig. S7: The legend doesn't explain that the lighter red lines are the results of individual ears.

*Author’s response to the comments: The sentence has been added in the legend: “The lighter red lines represent the ABR thresholds of individual injected ears”.*

- Fig. S8: The legend should clarify that mean +- SD are presented. Is the n=4 for panel B the sum of both groups (n=2 per group) or is the n=4 per group (clarified in panel A, but not panel B)?

*Author’s response to the comments: The sentence has been added in the legend: “Values and error bars reflect the mean ± SD in A and B”. For panel B, the n=4 is per group. We have revised the panel B for clarification.*

**Second round of review**

**Reviewer 2**

The authors satisfactorily addressed the comments, and the manuscript was much improved. Overall, these experiments are very interesting and relevant to the field of inner ear research.
